# Low‐Dose Staphylococcal Enterotoxin C2 Mutant Maintains Bone Homeostasis via Regulating Crosstalk between Bone Formation and Host T‐Cell Effector Immunity

**DOI:** 10.1002/advs.202300989

**Published:** 2023-08-08

**Authors:** Haixing Wang, Sien Lin, Lu Feng, Baozhen Huang, Xuan Lu, Zhengmeng Yang, Zhaowei Jiang, Yu‐Cong Li, Xiaoting Zhang, Ming Wang, Bin Wang, Lingchi Kong, Qi Pan, Shanshan Bai, Yuan Li, Yongkang Yang, Wayne Yuk Wai Lee, Peter D. Currie, Changshuang Lin, Yanfu Jiang, Juyu Chen, Micky D. Tortorella, Hongyi Li, Gang Li

**Affiliations:** ^1^ Musculoskeletal Research Laboratory, Department of Orthopaedics & Traumatology, Li Ka Shing Institute of Health Sciences The Chinese University of Hong Kong Hong Kong 999077 China; ^2^ Centre for Regenerative Medicine and Health Hong Kong Institute of Science & Innovation Chinese Academy of Sciences Hong Kong 999077 China; ^3^ Greater Bay Area Institute of Precision Medicine (Guangzhou) Fudan University 2nd Nanjiang Rd, Nansha District Guangzhou 511458 China; ^4^ Department of Orthopaedic Surgery Shanghai Jiao Tong University Affiliated Sixth People's Hospital Yishan Rd. 600 Shanghai 200233 China; ^5^ Department of Orthopaedics South China Hospital Shenzhen University Shenzhen 518116 China; ^6^ Australian Regenerative Medicine Institute Monash University Wellington Road Clayton Victoria 3800 Australia; ^7^ Shenyang Xiehe Biopharmaceutical Co. Ltd. Shenyang Liaoning Province 110179 China

**Keywords:** bone homeostasis, IFN‐γ, nitric oxide, staphylococcal enterotoxin C2, T cells

## Abstract

Studies in recent years have highlighted an elaborate crosstalk between T cells and bone cells, suggesting that T cells may be alternative therapeutic targets for the maintenance of bone homeostasis. Here, it is reported that systemic administration of low‐dose staphylococcal enterotoxin C2 (SEC2) 2M‐118, a form of mutant superantigen, dramatically alleviates ovariectomy (OVX)‐induced bone loss via modulating T cells. Specially, SEC2 2M‐118 treatment increases trabecular bone mass significantly via promoting bone formation in OVX mice. These beneficial effects are largely diminished in T‐cell‐deficient nude mice and can be rescued by T‐cell reconstruction. Neutralizing assays determine interferon gamma (IFN‐γ) as the key factor that mediates the beneficial effects of SEC2 2M‐118 on bone. Mechanistic studies demonstrate that IFN‐γ stimulates Janus kinase/signal transducer and activator of transcription (JAK–STAT) signaling, leading to enhanced production of nitric oxide, which further activates p38 mitogen‐activated protein kinase (MAPK) and Runt‐related transcription factor 2 (Runx2) signaling and promotes osteogenic differentiation. IFN‐γ also directly inhibits osteoclast differentiation, but this effect is counteracted by proabsorptive factors tumor necrosis factor alpha (TNF‐α) and interleukin 1 beta (IL‐1β) secreted from IFN‐γ‐stimulated macrophages. Taken together, this work provides clues for developing innovative approaches which target T cells for the prevention and treatment of osteoporosis.

## Graphical abstract

Bone marrow T cells stimulated by staphylococcal enterotoxin C2 (SEC2) 2M‐118 secrete interferon gamma (IFN‐γ) and nitric oxide (NO) into microenvironment. IFN‐γ increases NO production and activates p38 mitogen‐activated protein kinase (MAPK) signaling, which further upregulates Runt‐related transcription factor 2 (Runx2) and promotes bone formation. SEC2 2M‐118 also modulates bone resorption via factors secreted from T cells and macrophages.

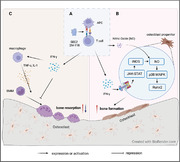

## 1 Introduction

Over the past decades, crosstalk between the immune and skeletal systems has been widely explored and demonstrated.^[^ ^1^, ^2^, ^3^, ^4^ ^]^ Deficiency or dysfunction of T cell or B cells can lead to imbalance of bone homeostasis,^[^ ^5^, ^6^, ^7^, ^8^ ^]^ indicating lymphocytes are essential stabilizers of basal bone turnover in vivo. More intriguingly, in vitro studies have also suggested that activated T cells have potent effects on osteogenesis and osteoclastogenesis. It has been reported that T cells activated by anti‐cluster of differentiation 3 (CD3) and anti‐CD28 antibodies significantly suppressed osteoclastogenesis in coculture assays.^[^ ^9^ ^]^ Moreover, conditioned medium obtained from cultures of activated T cells dramatically stimulated osteoblast differentiation.^[^ ^10^, ^11^ ^]^ These data suggest that T‐cell activators have the potential to influence bone homeostasis.

Compared with the potent effects of activated T cells on osteogenesis and osteoclastogenesis in vitro, the roles T cells play in bone homeostasis are much more complex in vivo, especially in pathological scenarios such as postmenopausal osteoporosis. Several studies have highlighted the role of T cells in estrogen‐deficiency‐induced bone loss.^[^ ^12^, ^13^, ^14^, ^15^, ^16^ ^]^ These studies suggested that the increasing proliferation and life span of T cells with enhanced production of tumor necrosis factor alpha (TNF‐α) was one essential mechanism for bone loss induced by estrogen deficiency. It was also showed that T‐cell‐deficient nude mice were protected from ovariectomy (OVX)‐ induced bone loss. However, this conclusion was soon challenged by other works,^[^ ^6^, ^17^ ^]^ which reported that T‐cell‐deficient mice lost trabecular bone equivalent to that of wild type mice. They also reported that the numbers and percentages of T cells in bone marrow were not increased after OVX. Thus, to date, it is not fully illuminated whether activated T cells are friends or foes to bone homeostasis. Moreover, whether administration of T‐cell activators in vivo may alleviate or aggravate estrogen deficiency‐induced bone loss is also uncertain.

Staphylococcal enterotoxins (SEs) are classical superantigens (SAgs) due to their potent capacity to activate T cells.^[^ ^18^, ^19^ ^]^ In contrast to normal antigens, SEs can directly bind to major histocompatibility complex class II (MHC II) molecules in a location adjacent to the peptide groove, activating T‐cell receptors (TCRs) by cross‐bridging with MHC II on antigen‐presenting cells (APCs).^[^ ^20^, ^21^, ^22^ ^]^ To date, nearly 25 serotypes of SEs have been identified.^[^ ^20^ ^]^ Among them, the type C SEs (SECs) are a group of highly conserved proteins that retain T‐cell stimulation ability but with lower toxicity, which makes it possible to develop SECs as a potential candidate for immunotherapy. In China, systemic administration of staphylococcal enterotoxin C2 (SEC2) (one subtype of SECs) has been approved by the Chinese National Food and Drug Administration for the treatment of malignant tumor and leukemia, and local injection for promoting bone fracture healing has been administered to patients since 1999. Our previous studies also revealed that SEC2 could promote bone regeneration, with local administration of SEC2 accelerating bone fracture healing and expediting bone consolidation in a distraction osteogenesis model.^[^ ^23^, ^24^ ^]^ These studies indicate a potential application of SEC2 for augmenting systemic bone formation. However, there is no report on the effect of systemic administration of SEC2 on bone homeostasis, and whether SEC2 could alleviate OVX‐induced bone loss remains to be tested.

In recent years, a mutant form of wild type SEC2 protein named SEC2 2M‐118 with reduced toxicity has been developed.^[^ ^25^ ^]^ The 2M‐118 has 3 point mutations at Thr20, Gly22, and His118. The substitution of histidine at position 118 has led to a reduction in adverse effects such as emesis and fever, whereas the point mutations at Thr20 and Gly22 improve superantigen activity by affecting MHC class II molecule binding and TCR binding.^[^ ^26^ ^]^ The development of this mutant SEC2 potentially extends its scope of clinical application with acceptable toxicity.

In this study, we applied systemic administration of SEC2 2M‐118 in OVX mice, which showed significant beneficial effects on maintaining bone homeostasis via factors secreted from T cells. We further identified interferon gamma (IFN‐γ) as the key factor that mediated the promoting effects of SEC2 2M‐118 on osteogenesis. Moreover, nitric oxide (NO)‐mediated activation of p38 mitogen‐activated protein kinase (MAPK) signaling and the downstream Runt‐related transcription factor 2 (Runx2) signaling were identified as the possible mechanisms underlying the effect of IFN‐γ on bone formation. These results provide insights for the development of new therapeutics which target T cells for the treatment of osteoporosis.

## 2 Results

### 2.1 Systemic Administration of SEC2 2M‐118 Alleviates Bone Loss in OVX Mice via Promoting Bone Formation

**Figure 1:**
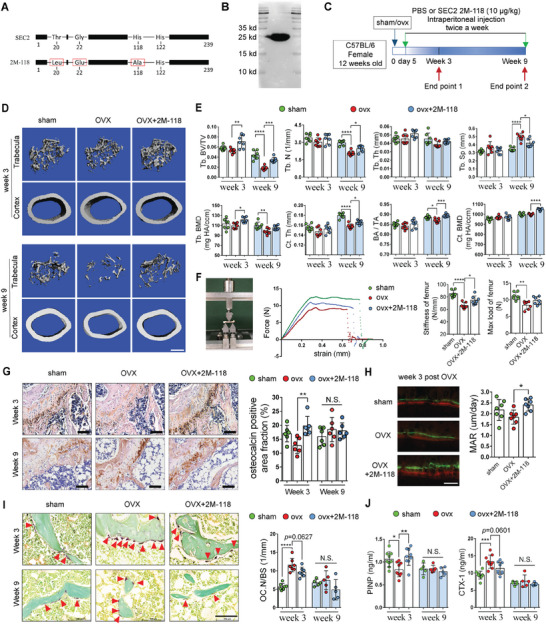
Systemic administration of SEC2 2M‐118 alleviates bone loss in OVX mice via promoting bone formation. A) Schematic diagram of SEC2 mutant protein 2M‐118. Red boxes indicate mutations compared with the wild type SEC2. B) The purified SEC2 2M‐118 was confirmed by Western blot. C) OVX mice received systemic administration of SEC2 2M‐118 and were terminated at two time points. D) 3D reconstruction of trabecular bone in distal femur and cortical bone in midshaft. Scale bar, 500 µm. E) Quantitative analyses of trabecular parameters (BV/TV, Tb. N, Tb. Th, Tb. Sp, and Tb. BMD) and cortical bone parameters (Ct. Th, BA/TA, Ct. BMD). *n* = 7. F) Three‐point bending test of femurs. Stiffness and max load of femurs were present. *n* = 7. G) Representative microphotographs and quantitative analyses of immunohistochemistry (IHC) staining for osteocalcin (OCN) from femur sections. Scale bar, 100 µm. *n* = 6. H) Representative images of calcein and xylenol orange double labeling of cortical bone in femur and quantification of mineral apposition rate (MAR). Scale bar, 50 µm. *n* = 7. I) Representative images of TRAP staining of osteoclasts and quantification of osteoclast number per bone surface (Oc. N/BS) in femur at weeks 3 and 9 post‐OVX‐surgery. The stained osteoclasts are indicated by arrows. Scale bar, 100 µm. *n* = 5–7. J) Serum levels of procollagen type I intact N‐terminal propeptide (PINP) and C‐terminal telopeptide of type 1 collagen (CTX‐1) at weeks 3 and 9 post‐OVX‐surgery. *n* = 5–9. Data were shown as mean ± Standard Deviation (SD). One‐way ANOVA was used with Bonferroni multiple comparisons test. * *p* < 0.05, ** *p* < 0.01, *** *p* < 0.001, **** *p* < 0.0001. N.S.: not significant.

SEC2 2M‐118 is a 25 kDa mutant protein derived from wild type SEC2 (3 point mutations at Thr20, Gly22, and His118) (**Figure** **1**A,B). To explore the effects of systemic administration of SEC2 2M‐118 on bone and evaluate its potential toxicity in vivo, we performed intraperitoneal injection of SEC2 2M‐118 with different dosages (100 ng kg^−1^, 1 µg kg^−1^, and 10 µg kg^−1^) in C57BL/6 mice (Figure S1A, Supporting Information). Micro‐computed‐tomography (micro‐CT) results showed the trabecular bone volume relative to total tissue volume (BV/TV) in distal femur was significantly increased in the SEC2 2M‐118 group at the dosage of 10 µg kg^−1^ (Figure S1B,C, Supporting Information). SEC2 2M‐118 also increased the trabecular bone mineral density (Tb. BMD) at the distal femur in a dose‐dependent manner (Figure S1B,C, Supporting Information). Body weight monitoring and histological examinations of major organs (heart, lung, liver, kidney, spleen, and intestine) indicated that there were no notable toxic effects when SEC2 2M‐118 was administrated systemically with the dosages up to 10 µg kg^−1^ (Figure S2, Supporting Information).

To further verify whether administration of SEC2 2M‐118 could alleviate estrogen‐deficiency‐induced bone loss, we established an OVX‐induced bone loss model using 12 weeks old C57BL/6 mice and monitored bone mass and bone turnover markers at weeks 3, 6, and 9 post‐OVX‐surgery. Micro‐CT results suggested that OVX‐induced bone loss was the most significant at week 9, while the imbalance of bone homeostasis was significant at week 3, according to the serum levels of bone turnover markers (Figure S3, Supporting Information). Thus, week 3 post‐OVX‐surgery was identified in our study as the optimal time point to evaluate bone remodeling status, while week 9 was chosen to determine the outcome of bone loss.

We then carried out the systemic administration of SEC2 2M‐118 (10 µg kg^−1^, intraperitoneal injection, twice a week) in the OVX mice (Figure 1C). Data from micro‐CT analysis showed that SEC2 2M‐118 treatment alleviated OVX‐induced trabecular bone loss in distal femur at week 9 (+88.65% in BV/TV, *p* < 0.001, comparing with the OVX control group) (Figure 1D,E). Meanwhile, significant protective effects of SEC2 2M‐118 on cortical bone were also shown in femur midshaft at week 9 (+5.07% in cortical bone thickness (Ct. Th), *p* < 0.05; +2.75% in bone area/tissue area (BA/TA), *p* < 0.001; +4.95% in BMD, *p* < 0.0001, comparing with the OVX control group) (Figure 1D,E). We also tested whether SEC2 2M‐118 treatment could rescue the deterioration of mechanical properties in the OVX mice. Results from three‐point bending tests showed that the maximum load and stiffness of femurs significantly decreased in the OVX group at week 9 (−24.47% in maximum load, *p* < 0.01; −22.31% in stiffness, *p* < 0.0001), while SEC2 2M‐118 treatment partially rescued the impaired mechanical properties with increased maximum load (+16.21%, *p* > 0.05, not significant) and stiffness (+12.91%, *p* < 0.05) (Figure 1F), indicating a beneficial effect of SEC2 2M‐118 treatment on cortical bone quality in the OVX mice.

We then investigated the effects of SEC2 2M‐118 treatment on bone remodeling. Immunohistochemistry (IHC) staining of bone formation marker osteocalcin (OCN), and calcein and xylenol orange double labeling all showed that SEC2 2M‐118 treatment effectively enhanced bone formation in the OVX mice at week 3 (Figure 1G,H), which was consistent with the Enzyme‐Linked Immunosorbent Assay (ELISA) results showing that serum levels of bone formation marker procollagen type I intact N‐terminal propeptide (PINP) were significantly enhanced by SEC2 2M‐118 treatment (+40.06% compared with OVX control group, *p* < 0.01) at week 3 post‐OVX (Figure 1J). We also detected bone resorption marker C‐terminal telopeptide of type 1 collagen (CTX‐1) and observed a significant increase of serum CTX‐1 in the OVX group at week 3 (+57.06% compared with the sham control group, *p* < 0.01), while SEC2 2M‐118 treatment slightly decreased serum levels of CTX‐1 (−14.52% compared with the OVX group, *p* = 0.0601) (Figure 1J). Moreover, tartrate‐resistant acid phosphatase (TRAP) staining in distal femurs also showed the osteoclast number per bone surface (Oc. N/BS) was slightly decreased by SEC2 2M‐118 treatment (−16.47% compared with the OVX control group, *p* = 0.0627) in OVX mice at week 3 (Figure 1I), but the effect was not statistically significant. These results indicate that the beneficial effects of SEC2 2M‐118 treatment on bone mass mainly result from its promoting effect on bone formation.

### 2.2 The Beneficial Effects of SEC2 2M‐118 Treatment on Bone Are Largely T‐Cell‐Dependent

**Figure 2:**
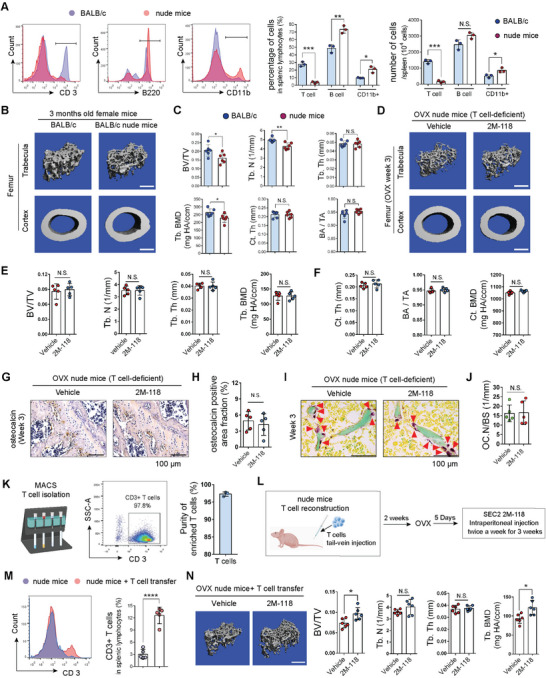
The beneficial effects of SEC2 2M‐118 treatment on bone are largely T‐cell‐dependent. A) Comparation of the proportion and number of splenic T cells, B cells, and CD11b+ cells between BALB/c mice and nude mice. B,C) Micro‐CT reconstruction and quantitative analyses of trabecular bone (BV/TV, Tb. N, Tb. Th, and Tb. BMD) and cortical bone (Ct. Th and BA/TA) in distal femurs of 3 months old female BALB/c mice and nude mice. Scale bar in (B), 500 µm. *n* = 6. D–F) Micro‐CT reconstruction and quantitative analyses of trabecular bone (E) and cortical bone (F) in distal femurs of OVX nude mice after SEC2 2M‐118 treatment for 3 weeks. Scale bar in (D), 500 µm. *n* = 5. G,H) Representative images and quantitative analyses of IHC staining of OCN from femur sections at week 3 post‐OVX‐surgery. Scale bar, 100 µm. *n* = 5. I,J) Representative images and quantitative analyses of TRAP staining of osteoclasts in femurs at week 3 post‐OVX‐surgery. The stained osteoclasts are indicated by arrows. Scale bar, 100 µm. *n* = 5. K) Pure T cells were enriched from splenocytes of 3 months old BALB/c mice. The purity of T cells was confirmed by flow cytometry. *n* = 3. L) 3 months old nude mice were subjected to adoptive transfer of wild type BALB/c splenic T cells, which was followed by OVX surgery and 2M‐118 treatment. M) Verification of T‐cell reconstruction in nude mice 2 weeks after T‐cell transfer. *n* = 5. N) Micro‐CT reconstruction and quantitative analyses of trabecular bone (BV/TV, Tb. N, Tb. Th, and Tb. BMD) in distal femurs of nude mice with T‐cell reconstruction after 2M‐118 treatment. Scale bar in (N), 500 µm. *n* = 6. Data were shown as mean ± SD. Student's *t*‐test was used to compare parameters between two groups. One‐way ANOVA with Bonferroni multiple comparisons test was used for multiple comparisons. * *p* < 0.05, ** *p* < 0.01, *** *p* < 0.001, **** *p* < 0.0001, N.S.: not significant.

To determine whether T cells were involved in the effects of SEC2 2M‐118 treatment on bone, we performed loss and gain of function assays using T‐cell‐deficient nude mice (BALB/c‐Foxn1^nu^/Arc). Flow cytometry analysis confirmed that there was only a very small proportion (about 2–3%) of T cells in splenocytes from the nude mice, while the percentages and numbers of B cells and CD11b+ cells (including monocytes, neutrophils, natural killer cells, granulocytes, and macrophages) were slightly higher in nude mice compared to BALB/c mice (**Figure** **2**A). Micro‐CT data also showed that 3 months old female nude mice exhibited decreased trabecular bone mass compared to the wild type controls (Figure 2B,C), indicating a potential role of T cells in maintaining bone homeostasis. More importantly, SEC2 2M‐118 treatment in OVX nude mice for 3 weeks showed no significant effect on most parameters regarding the trabecular bone (BV/TV, trabecular number (Tb. N), trabecular thickness (Tb. Th), and Tb. BMD) and cortical bone (Ct. Th, BA/TA, and Ct. BMD) (Figure 2D–F). We also examined the effects of SEC2 2M‐118 treatment on bone formation and resorption at week 3 post‐OVX‐surgery. IHC staining of OCN and TRAP staining of osteoclasts all showed no significant difference between SEC2 2M‐118 treatment group and the OVX control group in T‐cell‐deficient nude mice (Figure 2G–J). Then, we performed gain of function assay via T‐cell reconstruction in nude mice. Pure T cells were isolated from BALB/c wild type mice and transferred into T‐cell‐deficient nude mice (Figure 2K–M). Micro‐CT data showed the reconstruction of T cells in nude mice rescued the beneficial effects of 2M‐118 treatment on trabecular bone mass (Figure 2N). Collectively, these data suggested that the beneficial effects of SEC2 2M‐118 treatment on bone were largely T‐cell‐dependent.

### 2.3 SEC2 2M‐118 Promotes T‐Cell Proliferation and Activation

**Figure 3:**
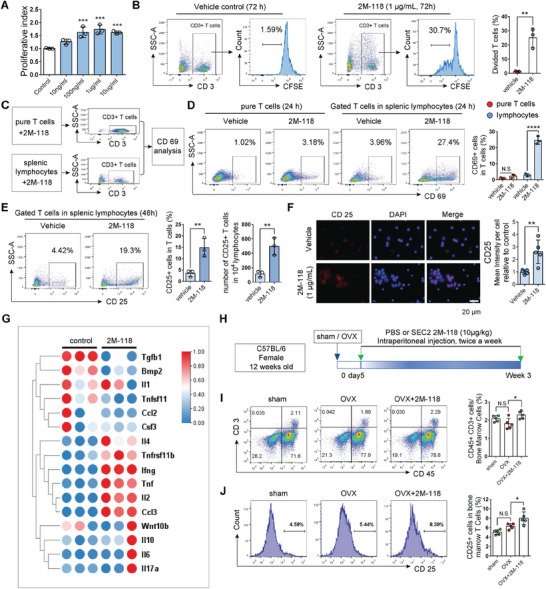
SEC2 2M‐118 promotes T‐cell proliferation and activation. A) Effect of SEC2 2M‐118 on the proliferation of splenic lymphocytes in vitro was determined by MTT assay after 3 days of stimulation. *n* = 3. B) Splenic lymphocytes were labeled with CFSE and treated with PBS or SEC2 2M‐118 (1 µg mL^−1^) for 3 days. The proportion of divided cells in T cells was determined by flow cytometry analysis. *n* = 3. C,D) Pure T cells or splenic lymphocytes were stimulated with or without SEC2 2M‐118 (1 µg mL^−1^) for 24 h. The activation of T cells was tested by evaluating CD69 using flow cytometry. *n* = 3. E) Proportion and absolute number of CD25+ T cells after SEC2 2M‐118 stimulation. *n* = 3. F) Representative images and quantitative analyses of immunofluorescent staining of CD25 in splenic lymphocytes after SEC2 2M‐118 (1 µg mL^−1^) stimulation. *n* = 5. G) Splenic lymphocytes were stimulated with SEC2 2M‐118 for 24 h. Total RNA was extracted. The expression levels of various secreted factors in 2M‐118 groups relative to control groups were presented by heatmap. H–J) OVX mice were treated with or without SEC2 2M‐118 for 3 weeks. The proportions of bone marrow total T cells (I) and CD25+ cells in total T cells (J) were analyzed by flow cytometry. *n* = 5. Data were shown as mean ± SD. Student's *t*‐test was used to compare parameters between two groups. One‐way ANOVA with Bonferroni multiple comparisons test was used for multiple comparisons. * *p* < 0.05, ** *p* < 0.01, *** *p* < 0.001, **** *p* < 0.0001. N.S.: not significant.

**Figure 4:**
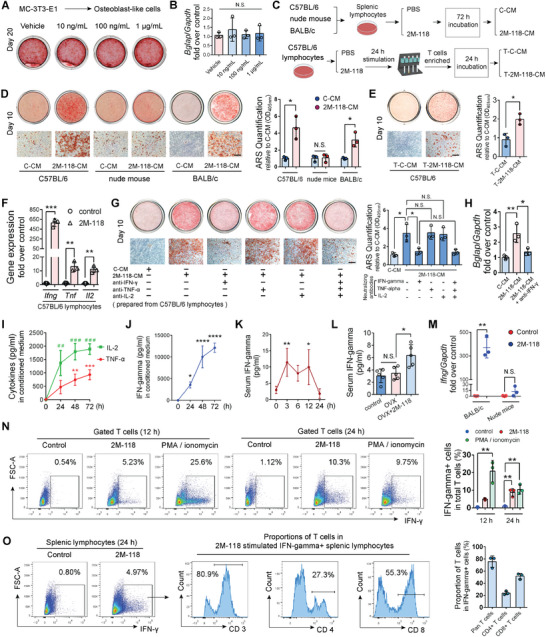
IFN‐γ is the key factor that mediates the promoting effect of SEC2 2M‐118 treatment on bone formation. A,B) Alizarin red staining (A) and RT‐qPCR analysis of *Bglap* (OCN) (B) showed various dosages of 2M‐118 exhibited no direct effects on osteogenic differentiation of MC‐3T3‐E1 cells. *n* = 3. C) Preparation of conditioned medium from splenic lymphocytes or enriched T cells was performed. The lymphocytes were obtained from C57BL/6 mice, BALB/c mice, or nude mice, while the enriched T cells were obtained from 2M‐118 prestimulated splenic lymphocytes (C57BL/6). D) MC‐3T3‐E1 cells were induced to differentiate into osteoblasts for 10 days. C‐CM (10%) or 2M‐118‐CM (10%) prepared from C57BL/6 mice, nude mice, or BALB/c mice was added into the differentiation medium. Quantification of Alizarin red staining showed the effects of 2M‐118‐CM on osteogenesis with or without the presence of T cells when conditioned medium was prepared. *n* = 3. Scale bar, 100 µm. E) Conditioned medium from enriched T cells (T‐C‐CM or T‐2M‐118‐CM, final concentration 20%) was added into the differentiation medium in osteogenesis assay. Quantification of Alizarin red staining showed the effects of T‐2M‐118‐CM on osteogenesis. *n* = 3. Scale bar, 100 µm. F) mRNA expression levels of *Ifng*, *Tnf*, and *Il2* in splenic lymphocytes after SEC2 2M‐118 stimulation. *n* = 3. G,H) Alizarin red staining (G) and RT‐qPCR (H) of *Bglap* (OCN) showed the effects of neutralization of specific cytokines (IFN‐γ, TNF‐α, and IL‐2) in 2M‐118‐CM on osteogenic differentiation and mineralization. *n* = 3. Scale bar in (G), 100 µm. I) Concentrations of IL‐2 and TNF‐α in 2M‐118‐CM collected at different time points. *n* = 3. J) Concentrations of IFN‐γ in 2M‐118‐CM collected at different time points. *n* = 3. K) Changes of serum IFN‐γ levels after the 1st exposure of SEC2 2M‐118 in C57BL/6 mice. *n* = 4–5. L) Serum IFN‐γ levels after the 6th exposure of SEC2 2M‐118 in OVX mice at week 3 post‐OVX‐surgery. *n* = 5. M) mRNA expression levels of *Ifng* in splenic lymphocytes from BALB/c mice or nude mice after SEC2 2M‐118 stimulation. *n* = 3. N,O) Splenic lymphocytes were stimulated with or without SEC2 2M‐118 (1 µg mL^−1^) for 12 or 24 h. The proportion of IFN‐γ secreting cells in T cells, and the percentages of T cells, CD4+ T cells, and CD8+ T cells in total IFN‐γ secreting cells were analyzed by flow cytometry. Phorbol 12‐myristate 13‐acetate (PMA)/ionomycin treatment was set as positive controls in (N), *n* = 3. Data were shown as mean ± SD. Student's *t*‐test was used to compare parameters between two groups. One‐way ANOVA with Bonferroni multiple comparisons test was used for multiple comparisons. * *p* < 0.05, ** *p* < 0.01, *** *p* < 0.001, **** *p* < 0.0001. N.S.: not significant.

As it was confirmed that T cells mediate the effects of SEC2 2M‐118 on bone, we further explored the influence of SEC2 2M‐118 on T‐cell proliferation and activation. Considering the activation of T cells needs costimulation signals from APCs, splenic lymphocytes which contain both T cells and APCs were used in this study, and pure T cells cocultured with or without irradiated APCs were also used as controls. First, we stimulated splenic lymphocytes with various dosages of SEC2 2M‐118 (10 ng mL^−1^, 100 ng mL^−1^, 1 µg mL^−1^, and 10 µg mL^−1^) in vitro for 3 days. MTT assay showed that SEC2 2M‐118 significantly promoted the proliferation of splenic lymphocytes at concentrations higher than 100 ng mL^−1^, and the concentration 1 µg mL^−1^ showed the maximal effect (**Figure** **3**A). Carboxyfluoroscein succinimidyl ester (CFSE) labeling assay further confirmed that T cells in splenic lymphocytes were proliferated upon the stimulation of SEC2 2M‐118 (Figure 3B). To evaluate the capacity of SEC2 2M‐118 to activate T cells, pure T cells, splenic lymphocytes, or pure T cells cocultured with irradiated APCs (Figure S4A, Supporting Information) were stimulated with or without SEC2 2M‐118 (1 µg mL^−1^) for 24 or 48 h. Analysis of T‐cell activation marker CD69 showed that SEC2 2M‐118 (1 µg mL^−1^) could not activate pure T cells efficiently, while T cells in splenic lymphocytes or pure T cells cocultured with irradiated APCs could be significantly activated by 2M‐118 (Figure 3C,D and Figure S4D (Supporting Information)). Flow cytometry analysis and immunofluorescent staining of CD25 also confirmed that T cells were activated after SEC2 2M‐118 stimulation (Figure 3E,F). We also collected messenger ribonucleic acid (mRNA) from splenocytes after SEC2 2M‐118 stimulation for 24 h in vitro and analyzed the expression levels of common cytokines. The mRNA expression levels of 4 cytokines, including interleukin 2 (IL‐2), TNF‐α, IFN‐γ, and C‐C motif chemokine ligand 3 (CCL3) were dramatically enhanced by more than tenfold (Figure 3G). Specially, IFN‐γ was the most upregulated cytokine which showed over 500‐fold increase (**Figure** **4**F). We further verified the in vivo effects of SEC2 2M‐118 treatment on bone marrow T cells. C57BL/6 female OVX mice (12 weeks old) were given systemic administration of SEC2 2M‐118 or phosphate‐buffered saline (PBS) for 3 weeks (Figure 3H). Flow cytometry data showed the proportions of bone marrow total T cells and CD25+ cells in T cells were both increased by SEC2 2M‐118 treatment (Figure 3I,J). These results indicate that SEC2 2M‐118 can effectively promote T‐cell proliferation and activation.

### 2.4 IFN‐γ Is the Key Factor That Mediates the Promoting Effect of SEC2 2M‐118 on Bone Formation

To identify whether T cells mediate the effect of SEC2 2M‐118 via secreted factors, we carried out in vitro osteogenesis assays using MC‐3T3‐E1 cells. First, we explored the direct effects of SEC2 2M‐118 on osteoblast differentiation. Alizarin Red S staining and quantitative reverse transcriptase polymerase chain reaction (RT‐qPCR) of *Bglap* (OCN) showed that various concentrations of SEC2 2M‐118 (10 ng mL^−1^, 100 ng mL^−1^, and 1 µg mL^−1^) had no significant effects on osteoblastic differentiation of MC‐3T3‐E1 cells (Figure 4A,B). We next prepared splenic lymphocytes and stimulated the cells with SEC2 2M‐118 (1 µg mL^−1^) or vehicle PBS for 3 days. The supernatant was collected as 2M‐118‐stimulated conditioned medium (2M‐118‐CM) or control conditioned medium (C‐CM) (Figure 4C). Interestingly, we found that 2M‐118‐CM prepared using splenic lymphocytes from C57BL/6 mice or BALB/c mice showed a remarkable enhancing effect on osteoblastic differentiation of MC‐3T3‐E1 cells, while 2M‐118‐CM prepared from T‐cell‐deficient nude mice had no or little effect (Figure 4D). More importantly, we also prepared conditioned medium of pure T cells enriched from 2M‐118‐stimulated splenic lymphocytes (Figure 4C), which were named as T‐2M‐118‐CM. A similar promoting effect of T‐2M‐118‐CM on osteogenesis was observed (Figure 4E), which strongly suggested that some specific secretory factors from T cells were involved in mediating the effects of SEC2 2M‐118 on bone formation.

To further identify the potential factors which mediate the effect of 2M‐118 on bone formation, we selectively neutralized the cytokines (IL‐2, TNF‐α, and IFN‐γ) which were significantly upregulated by 2M‐118 treatment (Figure 4F,I,J) to identify their effects on osteoblast differentiation. Alizarin Red S staining and RT‐qPCR analysis of *Bglap* showed that neutralization of IFN‐γ in 2M‐118‐CM largely abolished the promoting effect on osteogenic mineralization of MC‐3T3‐E1 cells (Figure 4G,H), whereas the neutralization of IL‐2 or TNF‐α showed no significant effects (Figure 4G), indicating that IFN‐γ was the potential key factor that mediated the promoting effect of SEC2 2M‐118 treatment on bone formation.

We then verified the protein levels of IFN‐γ after SEC2 2M‐118 treatment both in vitro and in vivo. ELISA results showed that the concentration of IFN‐γ in the conditioned medium kept increasing with the culture time and reached up to more than 10 000 pg mL^−1^ on day 3 (Figure 4J). We further determined the change of serum IFN‐γ levels after the exposure of SEC2 2M‐118 (10 µg kg^−1^) in C57BL/6 mice. After the first exposure of SEC2 2M‐118, the serum IFN‐γ levels were upregulated over twofold at 3 h, and then slowly decreased to normal levels at 24 h (Figure 4K). For OVX mice, we also observed that SEC2 2M‐118 exposure (6 times, 3 weeks) increased serum IFN‐γ levels 3 h after the last injection (Figure 4L). To verify whether the secretion of IFN‐γ after SEC2 2M‐118 treatment was T‐cell‐dependent, we repeated the SEC2 2M‐118 stimulation assay in vitro using splenic lymphocytes from nude mice or BALB/c mice. The absence of T cells dramatically abolished the promoting effect of SEC2 2M‐118 on IFN‐γ expression (Figure 4M). Furthermore, we found that the proportion of IFN‐γ secreting T cells was significantly increased after SEC2 2M‐118 stimulation for 24 h (Figure 4N). More importantly, flow cytometry analysis showed that T cells account for almost 80% of all IFN‐γ secreting splenic cells after 24 h of 2M‐118 stimulation, and the proportions of CD8+ T cells in IFN‐γ secreting cells were higher than CD4+ T cells (Figure 4O).

### 2.5 Nitric Oxide Mediates the Promoting Effect of IFN‐γ on Bone Formation via p38 MAPK–Runx2 Signaling

**Figure 5:**
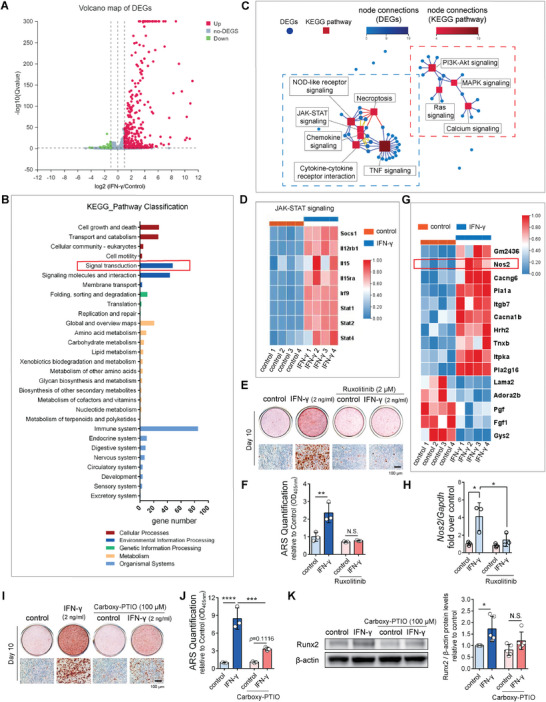
Nitric oxide mediates the promoting effect of IFN‐γ on bone formation. A) MC‐3T3‐E1 cells were induced to differentiate into osteoblasts with or without IFN‐γ (2 ng mL^−1^) for 7 days. Total RNA was collected for RNA‐sequencing analysis. A volcano map illustrating differentially expressed genes (DEGs) from RNA‐seq analysis between the control and IFN‐γ‐treated group. B) KEGG pathway classification analysis of all DEGs in (A). DEGs were grouped according to the following biological pathway: cellular processes; environmental information processing; genetic information processing; metabolism; organismal systems. C) KEGG pathway relationship network of the DEGs which were classified into signal transduction group in (B): the top 10 pathways with the largest number of genes were displayed. The blue dotted box and red dotted box highlighted two clusters of DEGs with close relationship. D) Heatmap of DEGs grouped into JAK–STAT signaling pathway presented in (C). E) Alizarin red staining showing the effect of JAK–STAT signaling inhibitor ruxolitinib (2 µm) on blocking the effect of IFN‐γ on osteoblast differentiation from MC‐3T3‐E1 cells. F) Quantification of Alizarin red staining in (E). *n* = 3. G) Heatmap of DEGs grouped into the cluster highlighted by red dotted box in (C). DEGs were ranked by fold change. H) Real‐time qPCR analysis of mRNA levels of *Nos2* in MC‐3T3‐E1 cells after IFN‐γ stimulation for 7 days with or without JAK–STAT signaling inhibitor ruxolitinib (2 µm). *n* = 3. I) Alizarin red staining showing the effects of IFN‐γ on osteoblast differentiation with or without the presence of NO scavenger carboxy‐PTIO (100 µm). J) Quantification of Alizarin red staining in (I). *n* = 3. K) Western blot analysis of protein expression levels of Runx2 in MC‐3T3‐E1 cells after IFN‐γ stimulation for 7 days with or without the presence of NO scavenger carboxy‐PTIO (100 µm). β‐actin served as loading control. *n* = 3. Data were shown as mean ± SD. One‐way ANOVA with Bonferroni multiple comparisons test was used for multiple comparisons. * *p* < 0.05, ** *p* < 0.01, *** *p* < 0.001, **** *p* < 0.0001. N.S.: not significant.

To further clarify what signaling pathways are potentially involved in the effect of IFN‐γ on bone formation, MC‐3T3‐E1 cells were induced for osteogenesis with or without the presence of IFN‐γ (2 ng mL^−1^) for 7 days and total RNA was extracted and sent for RNA‐sequencing analysis. 314 upregulated genes and 49 downregulated genes were identified with fold change ⩾ 2 and *Q* value ⩽ 0.05 (**Figure** **5**A). Then, all the differentially expressed genes (DEGs) were picked up for Kyoto Encyclopedia of Genes and Genomes (KEGG) pathway classification analysis. 48 DEGs were grouped into signal transduction group (Figure 5B). We further carried out KEGG pathway network analysis of these 48 DEGs (Figure 5C). The top 10 pathways with the largest number of DEGs were displayed and the potential connections between these pathways and DEGs were shown. We found that the DEGs were classified into two clusters: one cluster included JAK–STAT signaling, TNF signaling, and nucleotide oligomerization domain (NOD)‐like receptor signaling, etc., while the other cluster contained MAPK signaling, phosphoinositide 3‐kinase (PI3K)/Akt signaling, Ras signaling, and calcium signaling (Figure 5C). As it has been well proved that IFN‐γ can directly activate JAK–STAT pathway which leads to expression of various downstream genes,^[^ ^27^, ^28^ ^]^ we hypothesized that the cluster containing JAK–STAT signaling was the relative upstream signals, while the other cluster including MAPK signaling might be the downstream signals which further promote osteoblast differentiation. Then, we checked the DEGs belonging to JAK–STAT signaling and found all the DEGs were upregulated (Figure 5D). More importantly, the blockade of JAK–STAT signaling largely abolished the promoting effect of IFN‐γ on osteoblast differentiation (Figure 5E,F), indicating that JAK–STAT signaling was the critical upstream signals that mediated the effect of IFN‐γ on osteogenesis.

We next analyzed all the DEGs belonging to the other cluster (Figure 5C, highlighted by red dotted box). 10 DEGs were upregulated while 5 DEGs were downregulated (Figure 5G). Among them, nitric oxide synthase 2 (Nos2) (inducible nitric oxide synthase, iNOS) ranked second in terms of fold change. Moreover, we found the blocking of JAK–STAT signaling significantly attenuated the effect of IFN‐γ on promoting Nos2 (Figure 5H), suggesting that Nos2 is the downstream gene of JAK–STAT signaling. Considering that NO has been reported to play essential roles in osteoblast differentiation,^[^ ^29^ ^]^ we hypothesized that NO regulated by iNOS may be an important mediator of IFN‐γ on osteogenesis. We then repeated the IFN‐γ stimulation assay with the presence of NO radical scavenger carboxy‐PTIO (2‐(4‐carboxyphenyl)‐4,4,5,5‐tetramethylimidazoline‐1‐oxyl‐3‐oxide) (100 µm). Alizarin Red S staining and Western blot analysis of Runx2 revealed that carboxy‐PTIO treatment largely abolished the promoting effect of IFN‐γ on osteoblast differentiation (Figure 5I–K), suggesting that NO was an essential mediator of IFN‐γ.

**Figure 6:**
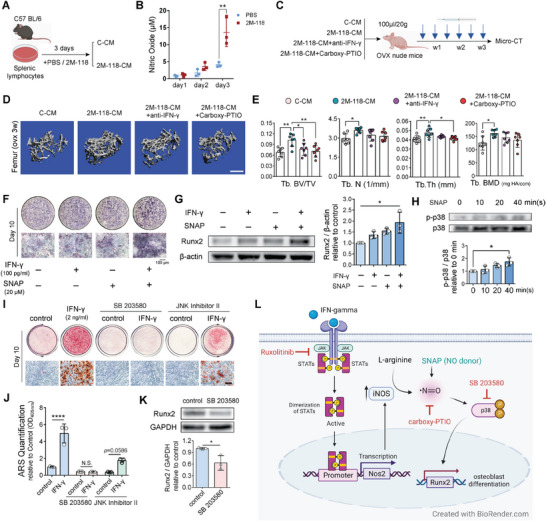
Nitric oxide magnifies the promoting effect of low‐dose IFN‐γ on osteoblast differentiation via p38 MAPK–Runx2 signaling. A) Preparation of conditioned medium using splenocytes from C57BL/6 mouse. B) The concentration of nitric oxide in the conditioned medium from days 1 to 3. *n* = 3. C–E) OVX nude mice received systemic administration of conditioned medium (100 µL per 20 g) prepared in (A) with or without the neutralization of IFN‐γ (10 µg mL^−1^ neutralizing antibody) or the scavenging of nitric oxide (carboxy‐PTIO, 1 mm in conditioned medium) for 3 weeks. 3D reconstruction and quantitative analyses of trabecular bone (BV/TV, Tb. N, Tb. Th, and Tb. BMD) in distal femurs of nude mice were shown in (D) and (E), respectively. *n* = 7. Scale bar in (D), 500 µm. F,G) IFN‐γ (100 pg mL^−1^), NO donor SNAP (20 µm), or a combination of both regents were added into the osteogenic induction medium of MC‐3T3‐E1 cells. ALP staining (F) and Western blot analysis of Runx2 (G) were performed on day 5. *n* = 3 in (G). H) Nitric oxide donor SNAP (100 µm) was added to MC‐3T3‐E1 cells for different times. Phosphorylation of p38 MAPK was determined and analyzed by Western blot. *n* = 3. I) Alizarin red staining showing the effects of p38 MAPK inhibitor SB 203580 (20 µm) and JNK inhibitor II (20 µm) on interfering the effect of IFN‐γ on osteoblast differentiation from MC‐3T3‐E1 cells. Scale bar, 100 µm. J) Quantification of Alizarin red staining in (I). *n* = 3. K) Western blot analysis of protein expression levels of Runx2 in MC‐3T3‐E1 cells after 5 days differentiation with or without the presence of p38 MAPK inhibitor SB 203580 (20 µm). *n* = 3. L) The schematic shows the proposed mechanism in which IFN‐γ promotes osteoblast differentiation. Data were shown as mean ± SD. Two‐way ANOVA with Bonferroni multiple comparisons test was used in (B). One‐way ANOVA was used with Bonferroni multiple comparisons test in (E), (G), (H), and (J). Student's *t*‐test was used in (K). * *p* < 0.05, ** *p* < 0.01, **** *p* < 0.0001. N.S.: not significant.

Considering that immune cells may be more sensitive in response to IFN‐γ and may be the major source of NO after IFN‐γ stimulation, we prepared 2M‐118‐CM using splenocytes from C57BL/6 mice (**Figure** **6**A) and detected the change of NO at different time points following SEC2 2M‐118 stimulation. We found that the concentration of NO increased significantly on day 3 (Figure 6B). To further verify the role of NO in mediating the promoting effect of SEC2 2M‐118 on bone formation in vivo, we injected the conditioned medium prepared from splenic lymphocytes (with or without IFN‐γ neutralizing antibody or carboxy‐PTIO) into OVX nude mice for 3 weeks (Figure 6C). Micro‐CT data showed that administration of 2M‐118‐CM significantly increased trabecular volume in T‐cell‐deficient nude mice, which mimicked the effect of SEC2 2M‐118 treatment in C57BL/6 mice (Figure 6D,E). More importantly, neutralization of IFN‐γ in 2M‐118‐CM or the administration of NO scavenger carboxy‐PTIO partially abolished the promoting effect of 2M‐118‐CM (Figure 6D,E). Inspired by these data, we further hypothesized that NO donor might magnify the promoting effect of low‐dose IFN‐γ on bone formation. We then stimulated MC‐3T3‐E1 cells with low‐dose IFN‐γ (100 pg mL^−1^), or NO donor *S*‐Nitroso‐*N*‐acetyl‐dl‐penicillamine (SNAP, 20 µm), or a combination of the two to see their effects on osteogenesis. Alkaline phosphatase (ALP) staining and Western blot analysis of Runx2 both suggested that supplement of NO donor enhanced the effect of low‐dose IFN‐γ on osteogenesis (Figure 6F,G). These data further determined that NO was involved in the effect of SEC2 2M‐118 on augmenting bone formation.

We next searched for the possible signaling pathway downstream of NO. It has been reported that NO production can upregulate p38 MAPK signaling,^[^ ^30^, ^31^ ^]^ which plays a critical role in modulating osteoblast differentiation. We also observed that NO donor SNAP stimulated the phosphorylation of p38 MAPK in MC‐3T3‐E1 cells (Figure 6H). More importantly, RNA‐sequencing analysis showed that the downstream of p38 MAPK signaling was activated after IFN‐γ stimulation, while c‐Jun N‐terminal kinase (JNK) MAPK and extracellular signal‐regulated kinase (ERK) signaling were not (Figure S6, Supporting Information). Furthermore, we found that blocking of p38 MAPK signaling using SB 203580 almost totally abolished the promoting effect of IFN‐γ on osteoblast mineralization (Figure 6I,J), indicating an essential role of p38 MAPK signaling in the effects of IFN‐γ. Western blot analysis also confirmed that suppression of p38 MAPK signaling inhibited Runx2 expression level (Figure 6K). Taken together, these results suggested that p38 MAPK–Runx2 signaling was the potential downstream of NO that mediated the promoting effect of IFN‐γ on bone formation (Figure 6L).

### 2.6 SEC2 2M‐118 Modulates Osteoclast Differentiation via Factors Secreted from T Cells and Macrophages

**Figure 7:**
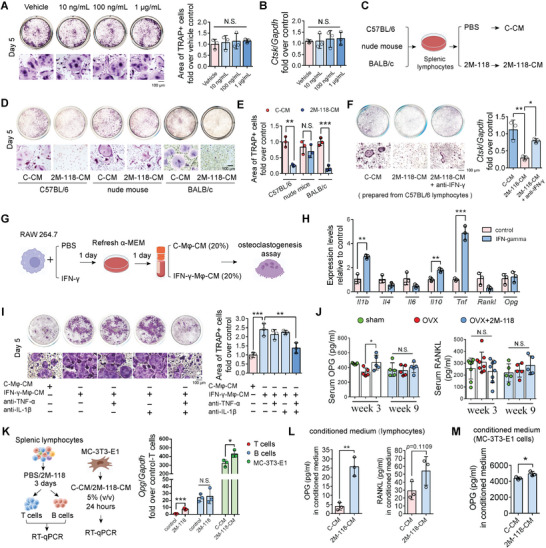
SEC2 2M‐118 modulates osteoclast differentiation via secreted factors from T cells and macrophages. A,B) TRAP staining (A) and RT‐qPCR analysis of cathepsin K (*Ctsk*) (B) showing the direct effects of SEC2 2M‐118 on osteoclastic differentiation from primary bone marrow monocytes. *n* = 3. C) Preparation of C‐CM and 2M‐118‐CM from splenic lymphocytes (C57BL/6 mice, nude mice, and BALB/c mice). D,E) Primary bone marrow monocytes were induced to differentiate into osteoclasts for 5 days. C‐CM (5%) or 2M‐118‐CM (5%) prepared from C57BL/6 mice, nude mice, or BALB/c mice were added into the differentiation medium. TRAP staining showed the effects of 2M‐118‐CM on osteoclastogenesis. *n* = 3. F) TRAP staining and RT‐qPCR analysis of *Ctsk* showed the neutralization of IFN‐γ in 2M‐118‐CM partially abolished its inhibiting effect on osteoclastogenesis. *n* = 3. G) RAW 264.7 cells were stimulated with IFN‐γ (2 ng mL^−1^) or PBS for 1 day. Culture medium was replenished, and supernatant was collected as IFN‐γ‐Mφ‐CM or C‐Mφ‐CM after another 24 h. Then, the collected conditioned medium was used for osteoclastogenesis at a concentration of 20%. H) RT‐qPCR analysis of various cytokines in RAW 264.7 cells with or without the stimulation of IFN‐γ (2 ng mL^−1^) for 12 h *n* = 3. I) TRAP staining showed the effects of C‐Mφ‐CM, IFN‐γ‐Mφ‐CM, and the neutralization of TNF‐α and IL‐1β in IFN‐γ‐Mφ‐CM on osteoclast differentiation. *n* = 3. J) Serum levels of OPG and RANKL after SEC2 2M‐118 treatment at weeks 3 and 9 post‐OVX‐surgery. *n* = 5–8. K) Splenic lymphocytes were treated with or without SEC2 2M‐118 (1 µg mL^−1^) for 3 days. T cells and B cells were isolated, and total RNA was extracted for RT‐qPCR analysis of *Opg* expression. MC‐3T3‐E1 cells were treated with C‐CM (5%) or 2M‐118‐CM (5%) for 24 h. The OPG expression levels were also analyzed by RT‐qPCR. *n* = 3. L) Splenic lymphocytes were treated with or without SEC2 2M‐118 (1 µg mL^−1^) for 3 days. The concentrations of OPG and RANKL in the conditioned medium were tested by ELISA. *n* = 3. M) MC‐3T3‐E1 cells were treated with C‐CM or 2M‐118‐CM for 24 h. Then, the culture medium was replenished with fresh α‐MEM for another 24 h. The conditioned medium was collected and concentration of OPG was evaluated. *n* = 3. Data were shown as mean ± SD. Student's *t*‐test was used to compare parameters between two groups in (H), (K), (L) and (M). One‐way ANOVA with Bonferroni multiple comparisons test was used for multiple comparisons in (A), (B), (E), (F), (I) and (J). * *p* < 0.05, ** *p* < 0.01, *** *p* < 0.001. N.S.: not significant.

To clarify the effect of SEC2 2M‐118 on bone resorption, we investigated the direct effect of SEC2 2M‐118 on osteoclast differentiation using primary bone marrow monocytes. TRAP staining and RT‐qPCR suggested that SEC2 2M‐118 had no significant direct effects on osteoclastic differentiation of primary bone marrow monocytes (**Figure** **7**A,B). Then, we further prepared 2M‐118‐CM using splenocytes from C57BL/6 mice, nude mice, or BALB/c mice (Figure 7C). 2M‐118‐CM prepared from C57BL/6 mice or BALB/c mice dramatically inhibited osteoclast differentiation, while the absence of T cells largely abolished this effect of 2M‐118‐CM (Figure 7D,E). Moreover, neutralization of IFN‐γ in 2M‐118‐CM also significantly attenuated its inhibiting effect on osteoclast differentiation (Figure 7F), suggesting that IFN‐γ in response to SEC2 2M‐118 treatment could directly inhibit osteoclast differentiation.

However, it should be noted that several studies reported that systemic administration of IFN‐γ led to enhanced bone resorption in vivo rather than inhibiting osteoclast differentiation.^[^ ^14^, ^32^ ^]^ We further stimulated RAW 264.7 cells with IFN‐γ or vehicle PBS for 24 h and refreshed the medium for another 24 h. The supernatant was collected as C‐Mφ‐CM or IFN‐γ‐Mφ‐CM (Figure 7G). RT‐qPCR analyses showed that IFN‐γ treatment significantly enhanced the expression of *Tnf* and *Il1b* in RAW 264.7 cells (Figure 7H), which have been well proved to promote osteoclast differentiation. Moreover, we found IFN‐γ‐Mφ‐CM promoted osteoclast differentiation from primary bone marrow monocytes, while the combined neutralization of TNF‐α and IL‐1β in IFN‐γ‐Mφ‐CM largely abolished this effect (Figure 7I). Taken together, these data suggested that IFN‐γ could indirectly promote osteoclast differentiation via secreted factors from macrophages.

In our study, we found that SEC2 2M‐118 treatment exhibited a trend to slightly inhibit bone resorption in vivo. Considering the net effect of in vivo administration of IFN‐γ is upregulation of bone resorption, we hypothesized that other factors may also mediate the effects of SEC2 2M‐118 on bone resorption. Then, we analyzed the expression of receptor activator of nuclear factor‐kappa B ligand (RANKL) and osteoprotegerin (OPG) (the two essential factors that regulate bone resorption in vivo) in response to SEC2 2M‐118 stimulation. We found that the serum levels of OPG were significantly upregulated by SEC2 2M‐118 treatment for 3 weeks after OVX surgery, while serum RANKL showed no significant difference (Figure 7J). RT‐qPCR analysis also showed that the expression of OPG in T cells (enriched from splenic lymphocytes after 2M‐118 stimulation) was significantly enhanced after SEC2 2M‐118 stimulation, while 2M‐118‐CM could slightly promote OPG expression in osteoblastic cells (Figure 7K). However, both the mRNA expression levels and secreted protein levels of OPG in splenic lymphocytes (T cells or B cells) were dramatically lower than osteoblastic cells (MC‐3T3‐E1) (Figure 7K–M), potentially indicating that the major source of the enhanced OPG expression upon SEC2 2M‐118 treatment is osteoblastic cells.

## 3 Discussion

Over the past decades, various drugs have been developed for osteoporosis management. To date, drugs such as bisphosphonates, selective estrogen‐receptor modulator, parathyroid hormone, and monoclonal antibodies to sclerostin (romosozumab) or RANKL (denosumab) have been used effectively in osteoporosis treatments.^[^ ^33^ ^]^ However, despite these remarkable advances, there are still many concerns about the side effects of antiresorptive drugs, particularly bisphosphonates.^[^ ^34^, ^35^ ^]^ Moreover, most of current available drugs belong to antiresorptive drugs, which are relatively ineffective in the treatment of osteoporotic fracture healing.^[^ ^36^ ^]^ Thus, developing new drugs with effective anabolic effects on bone is still highly warranted. Here, in this study, we show that systemic administration of SEC2 2M‐118 effectively alleviated OVX‐induced bone loss via its promoting effect on bone formation, and this effect was achieved with nonclassical mechanisms via T‐cell‐osteoblast crosstalk.

SEC2 is a heat‐stable enterotoxin that has potent capacity to stimulate T cells. As a mild SAg with low toxicity, SEC2 can efficiently activate cytotoxic T cells and induce various cytokines, which shows promising antitumor effects in vitro and in vivo.^[^ ^19^, ^26^ ^]^ Intriguingly, several studies, including our previous work, also showed that SEC2 significantly enhanced bone regeneration when applied locally.^[^ ^23^, ^24^, ^37^ ^]^ Here, our present work further shows that systemic administration of SEC2 2M‐118 in mice increased bone mass and maintained bone homeostasis, and the therapeutic effect was comparable with parathyroid hormone (PTH) or estradiol (E2) therapy in C57BL/6 mouse models.^[^ ^38^, ^39^ ^]^ More importantly, we demonstrated that the effect of SEC2 2M‐118 on bone was T‐cell‐dependent. First, as a kind of SAg, SEC2 2M‐118 can directly bind to TCR and MHC II molecules with high affinity.^[^ ^20^ ^]^ Thus, the stimulation of T cells is the major biological effect of SEC2 2M‐118 in vivo, and APCs without T cells could not respond to SEC2 2M‐118 stimulation efficiently (Figure 4M). Our in vitro osteogenesis assay also revealed that it was the secreted factors from T cells rather than the direct effect of SEC2 2M‐118 on osteoblast precursors that promoted osteogenesis. Besides, systemic administration of 2M‐118‐CM prepared from C57BL/6 mice in our study exhibited significant effect on augmenting bone mass in T‐cell‐deficient nude mice, which also confirmed the essential role of factors from lymphocytes on mediating the effects of SEC2 2M‐118 therapy. More importantly, our study demonstrated that the beneficial effect of SEC2 2M‐118 treatment was largely abolished in T‐cell‐deficient nude mice and could be rescued by T‐cell reconstruction, although the effects of SEC2 2M‐118 therapy in nude mice after T‐cell transfer were relatively weaker compared with wild type C57 BL/6 mice. One possible explanation is that the T‐cell reconstruction in nude mice is only a partial reconstruction (the T‐cell proportion in the splenic lymphocytes was around 12% in the reconstructed nude mice, whereas it was around 30% in the wild type mice). To avoid graft‐versus‐host effect, we can only transfer around 2 million allogenic T cells into nude mice. This may be the major reason why the beneficial effects of 2M‐118 therapy in nude mice after T‐cell transfer were not as strong as wild type mice. All the evidence above strongly suggests that T cells are involved in the beneficial effect of SEC2 2M‐118 on bone.

The crosstalk between T cells and bone cells is quite complex since various subsets of T cells show quite different and even opposite effects on bone remodeling.^[^ ^40^, ^41^, ^42^ ^]^ Thus, there are some discrepancies between published reports that define the role of T cells in OVX‐induced bone loss.^[^ ^6^, ^13^, ^16^, ^17^ ^]^ Moreover, studies also show controversial results about the change of T‐cell number/percentage in bone marrow after OVX surgery.^[^ ^6^, ^13^, ^43^, ^44^ ^]^ These discrepancies may result from variation in study designs regarding the age at which mice received OVX surgery and the choice of end point at which bone loss was evaluated.^[^ ^13^, ^38^, ^45^, ^46^ ^]^ Generally, a mature adult C57BL/6 mouse is considered 3–6 months old.^[^ ^47^ ^]^ This period represents a stage when development has ceased, but senescence has not yet started.^[^ ^47^ ^]^ To exclude the potential influence of developmental processes on bone metabolism,^[^ ^48^ ^]^ we chose 12 weeks old (3 months old) as the time point for OVX surgery (Figure S3, Supporting Information). Our study found a slight decrease of total T‐cell proportions in bone marrow at week 3 post‐OVX‐surgery, which was consistent with a published report,^[^ ^6^ ^]^ while SEC2 2M‐118 treatment effectively increased total T‐cell proportions, suggesting that expanded T‐cell population activated by SEC2 2M‐118 may potentially benefit bone homeostasis.

T cells can affect bone homeostasis either directly or indirectly via the secretion of various cytokines and factors.^[^ ^3^ ^]^ In accordance with previous report,^[^ ^10^ ^]^ our study also identifies IFN‐γ as the most essential factor that mediates the effects of activated T cells on bone formation. Interestingly, several clinical studies also identified IFN‐γ as a potential factor involved in primary osteoporosis. It has been reported that serum IFN‐γ levels in primary osteoporosis patients were significantly lower compared to health controls^[^ ^49^, ^50^ ^]^ and in vitro production of IFN‐γ by peripheral CD4+ T cells in OP women was also significantly lower.^[^ ^51^ ^]^ Over the past decades, the promoting effects of IFN‐γ on bone formation have been demonstrated by several studies,^[^ ^32^, ^52^, ^53^ ^]^ but the underlying mechanism has not been clearly elucidated. In general, IFN‐γ signaling leads to activation of various genes via the JAK–STAT pathway.^[^ ^27^, ^28^ ^]^ Individual IFN‐γ‐inducible genes can further activate various downstream targets, which finally lead to multiple effects on modulating inflammation, autoimmunity, cell differentiation, and apoptosis. Our study carried out bioinformatic analysis of IFN‐γ‐treated osteoblast‐like cells and identified NO as an essential mediator of IFN‐γ. The scavenging of NO largely abolished the promoting effect of IFN‐γ on bone formation in vitro and in vivo, which provides a new hint regarding how IFN‐γ promotes bone formation.

NO is a free radical which has important effects on bone remodeling.^[^ ^54^ ^]^ It has been previously shown that NO mediates the effects of estrogen on bone^[^ ^55^ ^]^ and the effects of mechanical loading on skeleton.^[^ ^56^ ^]^ Administration of pharmacological NO donors can increase bone mass and alleviate OVX‐induced bone loss.^[^ ^57^ ^]^ Moreover, NO has been demonstrated to activate p38 MAPK signaling,^[^ ^31^ ^]^ which potentially promotes the expression of Runx2. Our study also proved that NO magnified the promoting effect of low‐dose IFN‐γ on osteoblast differentiation via p38 MAPK–Runx2 signaling, which potentially explained why SEC2 2M‐118 treatment could show significant beneficial effects on bone with only a slight upregulation of IFN‐γ secretion in vivo.

Although the promoting effect of IFN‐γ on bone formation has been well documented, a few papers also reported a negative role of IFN‐γ on bone homeostasis under pathological conditions via the activation of bone resorption or the suppression of bone formation.^[^ ^14^, ^58^ ^]^ Our study also indicated that factors secreted from macrophages stimulated by IFN‐γ promoted osteoclast differentiation. These multiple “direct” and “indirect” effects (via macrophages) of IFN‐γ on bone cells suggest a complex role of IFN‐γ under different scenarios on bone homeostasis. Furthermore, the relatively high or low concentrations of IFN‐γ in the local microenvironment may also lead to different outcomes regarding its effects on bone, considering that high dosage of IFN‐γ may result in the activation of various immune cells such as macrophages, which in turn secretes proinflammatory cytokines which promote bone resorption. In our study, the dosage of SEC2 2M‐118 applied in vivo was low and we only detected a moderate enhancement of serum IFN‐γ levels after SEC2 2M‐118 treatment, which possibly explained why no obvious activation of bone resorption was observed in our study. Interestingly, we also observed an upregulation of serum OPG (a soluble decoy receptor for RANKL) levels after SEC2 2M‐118 treatment at week 3 post‐OVX, which potentially led to the slight suppression of bone resorption by SEC2 2M‐118 treatment.

Last but not the least, the safety of SEC2 2M‐118 for systemic administration is of great concern. In China, the SEC2 injection (10 ng mL, 4–12 mL for intravenous injection once a day) has been applied clinically with proven safety and efficacy for more than 28 years for the treatment of malignant tumor and leukemia. Several animal studies using rodents also demonstrated that the systemic administration of SEC2 in mice (15 µg per mouse, injected via tail vein every 2 days for 14 days) showed no obvious toxicity.^[^ ^26^ ^]^ It should be noted that the dosages of SEC2 in these studies were much higher than the dosage applied in the clinical practice. One important reason is the big difference of the affinity of SEs for the MHC II molecules expressed on APCs between humans and rodents. It was reported that the affinity of staphylococcal enterotoxin A (SEA) for the MHC II molecules in humans was nearly 100‐fold higher than its affinity in rodents.^[^ ^59^ ^]^ Thus, to achieve similar immune responses, the dosages of SEs for mice in our study were much higher than those applied in humans. Nevertheless, no notable toxicity was observed in our study via monitoring body weight and examining the morphological changes of major organs, suggesting that the dosages up to 10 µg kg^−1^ (about 0.25 µg per mouse) are relatively safe for systemic administration of SEC2 2M‐118. However, from a translational point of view, more preclinical studies are needed to test the safety and efficacy of SEC2 2M‐118 in a wider range of dosage.

In conclusion, the present study reported a novel application of mutant SEC2 2M‐118 on maintaining bone homeostasis in OVX mice. The anabolic effect of SEC2 2M‐118 treatment resulting from T‐cell‐derived IFN‐γ, and JAK/STAT–NO–p38 MAPK–Runx2 signaling pathway is the potential mechanism for the promotion of bone formation. The current work provides evidence and insights for targeting T cells as a potential new therapeutic approach for osteoporosis treatment. Nevertheless, more investigations are needed to verify our findings in large animal models and clinical trials. Future investigations into the crosstalk between immune and skeletal systems may foster the development of novel treatment strategies for bone metabolic disorders such as osteoporosis.

## 4 Experimental Section

### Animal Models

All animal experimental protocols were approved by the Animal Experimental Ethical Committee of the Chinese University of Hong Kong (CUHK) (Approval No. 19/213/MIS). 10–12 weeks old female C57BL/6 mice, BALB/c mice, athymic BALB/c nude mice (BALB/c‐Foxn1^nu^/Arc), or SD rats were purchased from the animal center of the CUHK. The mice were maintained under controlled temperature around 20 °C with a 12 h light/12 h dark cycle with free access to water and a pelleted commercial diet. Ovariectomized mouse model was established as followings: 12 weeks old female C57BL/6 mice, or nude mice under general anesthesia and sterile condition were used. The sham surgery was carried out with the extraction of the surrounding fatty tissue of bilateral ovaries, leaving the ovaries intact, whereas bilateral OVX for mice involved the full removal of both the left and right ovaries.

### Micro‐Computed‐Tomography Scanning

The structural changes of femurs were analyzed using micro‐CT (Scanco Medical, Switzerland). Briefly, the right femurs were fixed in a custom‐made holder. Image acquisition was performed at 70 kV and 118 µA, with a resolution of 10 µm per voxel. The grayscale images were segmented to perform 3D reconstruction of the mineralized bone phase to quantify microarchitecture of trabecular and cortical bone using the software of the micro‐CT workstation. For trabecular bone analysis, 100 continuous slices located under the growth plate inside the cortical bone were selected as the volume of interest (VOI). BV/TV, Tb. Th, Tb. N, trabecular separation (Tb. Sp), and BMD were analyzed using the built‐in program (Image Processing Language v4.29d, Scanco Medical). For cortical bone assessment, 100 continuous slices of the cortical bone in the midshaft of femurs were selected as the VOI. Ct. Th, BA/TA, and BMD were analyzed.

### Three‐Point Bending Mechanical Test

The left femurs were harvested for mechanical test. A three‐point bending device (H25KS, Hounsfield Test Equipment Ltd., UK) with 50 N load cell was used. The femurs were placed in the anterior–posterior direction with the two blades set at a span of 10 mm. The force loading point was set at the midshaft of femur, and the long axis of femur was placed perpendicular to the blades during the test. The Young's modulus (also known as the stiffness) and ultimate load were obtained for analysis and comparison.

### In Vivo Double Labeling of Bone Mineralization

Calcein (10 mg kg^−1^) and xylenol orange (90 mg kg^−1^) (both from Sigma‐Aldrich, St. Louis, MO, USA) were injected subcutaneously on days 13 and 3 before sacrifice, respectively. After termination, femurs were fixed in 4% paraformaldehyde solution, followed by dehydration using gradient ethanol, vitrification by xylene and embedded in methyl methacrylate. The bones were cut coronally into 10 µm thick sections with the RM2155 hard tissue microtome (Leica, Wetzlar, Germany). The double labeling images of bone slices were captured using a fluorescent microscope (Leica DM5500 system, Germany). Cortical bone formation was analyzed in four randomly selected visual fields in the midshaft of femur. Mineral apposition rate (MAR) was analyzed by OsteoMeasure Image Analysis System (Osteometrics, Decatur, GA, USA).

### Immunohistochemistry and Immunofluorescence Staining

The dewaxed slides were treated with citrate buffer (10 mm citric acid, pH 6.0) for 1 h at 70 °C to unmask antigen. Sections for immunohistochemistry were treated with 3% hydrogen peroxide for 20 min for the elimination of endogenous peroxidase activity. After blocking with 1% goat serum, the slides were incubated with rabbit anti‐OCN (1:100, Santa Cruz Biotechnology, CA, USA) antibody overnight at 4 °C. The slides were then incubated with goat anti‐rabbit secondary antibody and visualized using diaminobenzidine staining kit. For immunofluorescent staining of CD25, the slides were incubated with mouse anti‐CD25 antibody (1:50, Invitrogen, USA). Secondary reactions were done using Alexa‐Fluor 594‐conjugated anti‐mouse immunoglobulin G (IgG) secondary antibody (Thermo Fisher Scientific). Slides were then washed and mounted with ProLong Gold antifade reagent which contains 4',6‐diamidino‐2‐phenylindole (DAPI) (Thermo Fisher Scientific). Immunofluorescent images were captured using a fluorescent microscope (Leica DM5500 system, Germany).

### ELISA

Blood samples were put at room temperature to clot for 2 h before centrifuging for 10 min at 2000 *g*. The resultant sera were stored at −80 °C until analysis. The levels of PINP (BlueGene Biotech, Shanghai, China), CTX‐1 (Immunodiagnostic Systems, UK), IFN‐γ (R&D Systems, MN, USA), TNF‐α and IL‐2 (ABclonal Technology, Wuhan, China), RANKL and OPG (BlueGene Biotech, Shanghai, China) in serum or conditioned medium were detected according to the manufacturer's instructions.

### Histological TRAP Staining

The right femurs were decalcified at room temperature in 10% buffered ethylenediaminetetraacetic acid for 2 weeks. Then, the bones were dehydrated using gradient ethanol, vitrified by xylene, and embedded in paraffin. 5 µm thick sections were prepared with a rotary microtome (RM2255; Leica Microsystems, Germany), then followed by TRAP staining and counterstained with Fast Green. For the analysis of osteoclasts, five fields of view in the distal femur were randomly selected for each bone slice. The Oc. N/BS, mm^−1^ was analyzed using an OsteoMeasure Image Analysis System (Osteometrics, Decatur, GA, USA).

### Cell Purification

Splenic total T cells and B cells were enriched by magnetic‐activated cell sorting (MACS) using the following kits from Miltenyi Biotec (mouse Pan T Cell Isolation Kit, mouse Pan B Cell Isolation Kit) according to the manufacturer's instructions. The purity of cells was confirmed by flow cytometry analysis following cell isolation.

### Adoptive Transfer of T Cells

3 months old BALB/c nude mice were subjected to adoptive transfer of wild type (WT) BALB/c spleen T cells via tail‐vein injection of 2 × 10^6^ T cells purified by negative immunomagnetic selection (mouse Pan T Cell Isolation Kit, Miltenyi Biotec, Auburn, CA). T cells were transferred into nude mice 2 weeks before OVX surgery and 2M‐118 treatment to allow the peripheral expansion of the transferred T cells.

### Splenocyte Proliferation Assay

Splenocytes from 8 weeks old C57BL/6 female mice were used for the methylthiazolyldiphenyl‐tetrazolium bromide (MTT) assay to determine the effects of SEC2 2M‐118 on the proliferation of splenocytes. Briefly, splenocytes were seeded into 96‐well plates at a density of 8 × 10^5^ cells per well in Roswell Park Memorial Institute (RPMI) 1640 medium containing 10% fetal bovine serum (FBS). Serial tenfold dilutions of SEC2 2M‐118 in PBS were added to each well, starting from 10 µg mL^−1^ and with dilution to 10 ng mL^−1^. PBS served as the negative control. The cells were cultured at 37 °C with 5% CO_2_ for 72 h. Then, 10 µL of 5 mg mL^−1^ MTT dissolved in PBS was added to each well, followed by another 4 h incubation at 37 °C. Then, the cells were collected by centrifugation, and the cell pellet was resuspended in 120 µL dimethyl sulfoxide at room temperature for 10 min. The optical density (OD) was determined using a microplate reader set to 570 nm wavelength (OD570), with a reference wavelength set at 630 nm (OD630), and the final absorbance value was the difference between OD570 and OD630. The proliferation effect was reported as proliferation index (PI), PI = Abs value in experimental groups/Abs value in negative control groups.

### CFSE Cell Division

CFSE labeling was performed as follows: splenic lymphocytes isolated by Ficoll gradient were washed with PBS and incubated with 1 µm CFSE (Invitrogen, USA) in PBS for 8 min at 37 °C. Then, the labeling was stopped by adding equal volume pure FBS for 10 min. Cells were then washed with medium and seeded into 96‐well plates at a density of 2 × 10^6^ cells per well in RPMI 1640 medium containing 10% FBS with or without the stimulation of 1 µg mL^−1^ SEC2 2M‐118 for 3 days. The change of CFSE labeling was analyzed by flow cytometry.

### Cell Irradiation

T‐cell‐depleted splenic cells were isolated by MACS (mouse Pan T Cell Isolation Kit, Miltenyi Biotec) and then received gamma irradiation (100 Gy) in GC 3000 irradiator (MDS Nordion, Ottawa, ONT).

### T‐Cell Activation Assay

Splenic lymphocytes, pure T cells, or pure T cells with irradiated APCs (ratio of T cells to APCs, 1:1) were seeded into 96‐well plates at a density of 1 × 10^6^ cells per well in RPMI 1640 medium containing 10% FBS with or without the stimulation of 1 µg mL^−1^ SEC2 2M‐118 for 24 or 48 h. T‐cell activation markers CD69 and CD25 were analyzed by flow cytometry.

### Flow Cytometry Analysis

Splenic lymphocytes or bone marrow cells were washed thoroughly and filtered before being stained with directly conjugated antibodies for 30 min on ice. To analyze the proportion of T cells, B cells, and CD11b+ cells in splenic lymphocytes from BALB/c mice and nude mice, cells were stained with BV510 anti‐CD3e (BD Biosciences, San Jose, CA, USA), fluorescein isothiocyanate (FITC) anti‐B220 (BD Biosciences, San Jose, CA, USA), or Alexa Fluor 488 anti‐CD11b+ (BioLegend, CA, USA), respectively. To analyze total T cells in bone marrow, cells were stained with FITC anti‐CD45 (BD Biosciences, San Jose, CA, USA) and BV510 anti‐CD3e (BD Biosciences, San Jose, CA, USA). To determine the activation of T cells, cells were stained with APC anti‐CD3e (Invitrogen, Carlsbad, USA), FITC anti‐CD69 (Invitrogen, Carlsbad, USA), and PE anti‐CD25 (BD Biosciences, San Jose, CA, USA). To determine the proportion of IFN‐γ secretory cells in splenic lymphocytes, the cells were stimulated with 1 µg mL^−1^ SEC2 2M‐118 for 12 or 24 h. GolgiStop Protein Transport Inhibitor (BD Biosciences, San Jose, CA, USA, 0.6 µL mL^−1^) was added into the culture medium 6 h before the collection of the cells. Then, the cells were stained with APC anti‐CD3e (Invitrogen, Carlsbad, USA), FITC anti‐CD4 (BD Biosciences, San Jose, CA, USA), or APC–Cy7 anti‐CD8a (BD Biosciences, San Jose, CA, USA), which followed intracellular staining of BV421 anti‐IFN‐ γ (BD Biosciences, San Jose, CA, USA). After all the staining, cells were washed, filtered, and then detected by flow cytometry on BD LSRFortessa (BD Biosciences, San Jose, CA, USA), and the data were analyzed using FlowJo (Tree Star, Ashland, USA) software. Results were presented as cell frequencies.

### In Vitro Osteogenesis Assay

MC‐3T3‐E1 cells (ATCC CRL‐2593, Manassas, VA, USA) or rat BMSCs were cultured in alpha modified Eagle's medium (α‐MEM, Invitrogen, USA) supplemented with 10% FBS, (Gibco, USA), 100 U mL^−1^ penicillin, and 100 mg mL^−1^ streptomycin at 37 °C with 5% CO_2_. Osteogenic differentiation was performed as follows: briefly, MC‐3T3‐E1 cells or rat bone marrow‐derived mesenchymal stem cells (BMSCs) were seeded into a 24‐well plate at a density of 2 × 10^5^ cells mL^−1^. When over 80% confluence was reached, the medium was removed and replaced by the osteogenic induction medium (OIM, 50 µm l‐ascorbic acid‐2‐phosphate and 10 mm β‐glycerophosphate with complete medium). To investigate the potential roles of special signaling pathways in osteogenesis, the following chemicals were added into the OIM: ruxolitinib (2 µm, MedChemExpress, USA), carboxy‐PTIO (100 µm, MedChemExpress, USA), SNAP (20–100 µm, Sigma‐Aldrich, St. Louis, MO), SB 203580 (20 µm, Sigma‐Aldrich, St. Louis, MO), and JNK Inhibitor II (10 µm, Sigma‐Aldrich, St. Louis, MO). Alizarin Red staining or ALP staining was performed to clarify the effects of various treatments on osteogenic differentiation and the RNAs were harvested from the cells to determine the osteogenic gene expression profiles.

### Preparation of Conditioned Medium

For the preparation of conditioned medium from splenic lymphocytes, spleens from 8 weeks old C57BL/6 mice, nude mice, or BALB/c mice were used. Briefly, spleens were minced by the flat end of plungers from sterile 3 cc syringes in the culture medium to prepare single cell suspensions. Then, splenic lymphocytes were isolated by Ficoll‐Paque density gradient centrifugation according to the manufacturer's instructions (Ficoll‐Paque PLUS, density 1.077 ± 0.001 g mL^−1^, GE Healthcare, Sweden). The isolated lymphocytes were seeded into the wells of 6‐well plates at a density of 8 × 10^6^ cells mL^−1^ in RPMI 1640 medium containing 10% FBS. SEC2 2M‐118 was added into the culture medium at a concentration of 1 µg mL^−1^, and PBS served as vehicle control. The cells were cultured at 37 °C with 5% CO_2_ for 72 h. Then, the cell suspensions were harvested, centrifuged, and the supernatant was collected as conditioned medium. Specially, 2M‐118‐CM (containing 10–15 ng mL^−1^ IFN‐ γ and 10–20 µm nitric oxide) was called as a short form of the conditioned medium from lymphocytes stimulated by SEC2 2M‐118, while the conditioned medium from the resting control lymphocytes was defined as C‐CM. For the preparation of conditioned medium from purified T cells, the T cells enriched from 2M‐118 pretreated splenocytes were seeded into the wells of 24‐well plates at a density of 1 × 10^7^ cells mL^−1^ in RPMI 1640 medium containing 10% FBS for 24 h. Then, the cell suspensions were harvested, centrifuged, and the supernatant was collected as T‐C‐CM or T‐2M‐118‐CM. In osteogenesis assay, the conditioned medium was added into the differentiation medium at a ratio of 10% or 20% v/v, while for osteoclastogenesis assay, 5% volume conditioned medium was used. Fresh conditioned medium was added at each medium change. For the preparation of conditioned medium from macrophages, RAW 264.7 cells (ATCC TIB71, Manassas, VA, USA) were used. Briefly, RAW 264.7 cells were seeded into the wells of 6‐well plates at a density of 2 × 10^5^ cells mL^−1^ in α‐MEM containing 10% FBS. IFN‐γ (2 ng mL^−1^) or PBS was added into the culture medium for 24 h. Then, the culture medium was replenished with fresh α‐MEM. After another 24 h, the medium was collected, filtered, and stored at −20 °C as conditioned medium. Specially, conditioned medium from RAW 264.7 cells stimulated with IFN‐γ was defined as IFN‐γ‐Mφ‐CM, while the control conditioned medium was defined as C‐Mφ‐CM. In osteoclastogenesis assay, the conditioned medium was added into the differentiation medium at a concentration of 20%, and fresh conditioned medium was added at each medium change.

### Antibody Neutralization of Specific Cytokines in Conditioned Medium

To neutralize the activity of specific cytokines, 2M‐118‐CM or IFN‐γ‐Mφ‐CM was first diluted at 1:10 with osteogenic induction medium or osteoclast induction medium, then the conditioned medium was pretreated with the following antibodies (all purchased from R&D systems, MN, USA) for 1 h at 37 °C, respectively, before being added into the culture medium: rat anti‐mouse IFN‐γ (10 µ/mL^−1^), goat anti‐mouse TNF‐α (5 µg mL^−1^), goat anti‐mouse IL‐2 (5 µg mL^−1^) and hamster anti‐mouse IL‐1β (5 µg mL^−1^). The used concentrations of the antibodies were sufficient to neutralize more than 10 ng mL^−1^ IFN‐γ, 5 ng mL^−1^ TNF‐α, 5 ng mL^−1^ IL‐2, and 5 ng mL^−1^ IL‐1β according to the manufacturer's instructions.

### RNA Extraction and Real‐Time Quantitative PCR Analysis

Total RNA was extracted using TRIzol regent (Invitrogen, Carlsbad, CA, USA). The amount of RNA was measured using NanoDrop 2000 (ND‐2000; Thermo Scientific, USA) and complementary DNA (cDNA) synthesis was performed with 500 ng RNA using reverse transcriptase (TaKaRa Biotechnology, Otsu, Japan). Quantitative real‐time PCR was performed with SYBR Green Master Mix (Thermo Fisher, Waltham, USA) using an ABI 7500 Sequencing Detection System (Applied Biosystems, Foster City, CA, USA). Relative levels of gene expression were calculated by 2^−ΔΔCt^ method using Glyceraldehyde‐3‐phosphate dehydrogenase (GAPDH) as an endogenous control. The specific primer sequences are described in Table S1 (Supporting Information).

### RNA Sequencing and Bioinformatic Analysis

MC‐3T3‐E1 cells were induced for osteogenesis with or without IFN‐γ stimulation for 7 days and total RNA was extracted using TRIzol regent (Invitrogen, Carlsbad, USA) following the manufacturer's protocol. Total RNA was submitted to The Beijing Genomics Institute (BGI) TECH SOLUTIONS (HONGKONG) CO., LIMITED for RNA sequencing. DEGs were identified from RNA‐seq data with DESeq2.^[^ ^60^ ^]^ DEGs were defined as fold change ⩾ 2 and *Q* value ⩽ 0.05. KEGG pathway analyses were carried out based on the DR.TOM system of BGI.

### Western Blot Analysis

Cells were lysed by radioimmunoprecipitation assay (RIPA) Buffer (Sigma‐Aldrich, St. Louis, MO) containing protease inhibitors and phosphatase inhibitors. Lysates were subjected to centrifugation at 12 000 *g* at 4 °C for 20 min. The supernatants were collected, and protein concentrations were determined by bicinchoninic acid (BCA) Protein Assay Kit (Thermo Scientific, USA). Heat‐denatured proteins were separated by sodium dodecyl sulfate polyacrylamide gel electrophoresis (8–10%) and transferred onto polyvinylidene fluoride (PVDF) membranes. Membranes were blocked for 30 min in Tris‐buffered saline containing 5% skim milk at room temperature and probed with the following primary antibodies overnight at 4 °C: Runx2 (Cell Signaling Technology, 1:1000), β‐actin (Santa Cruz Biotechnology, 1:2000), GAPDH (Invitrogen, 1:10 000), phospho‐p38 MAPK (Cell Signaling Technology, 1:1000), and total p38 MAPK (Cell Signaling Technology, 1:1000). Then, membranes were washed and incubated with the appropriate horseradish‐peroxidase‐conjugated secondary antibodies for 2 h at room temperature. Protein bands were detected using enhanced chemiluminescence reagents (GE Healthcare, Buckinghamshire, UK) and analyzed using the Image J software (National Institutes of Health, Bethesda, MD, USA).

### In Vitro Osteoclastogenesis Assay

Primary mouse bone marrow monocytes (BMMs) were differentiated into osteoclasts. Briefly, whole bone marrow cells were flushed from the femurs of 3 months old female C57BL/6 mouse and plated in 100 mm culture plates in α‐MEM supplemented with 10% FBS and 1% penicillin/streptomycin for 24 h. Nonadherent cells were collected and seeded in 24‐well plates at a density of 2 × 10^5^ cells per well in α‐MEM supplemented with 10% FBS, 1% penicillin/streptomycin, and 30 ng mL^−1^ recombinant macrophage colony‐stimulating factor (M‐CSF) (R&D Systems, MN, USA) for 3 days. Then, the BMMs were incubated with OC) differentiation medium containing 30 ng mL^−1^ recombinant M‐CSF and 30 ng mL^−1^ recombinant RANKL. Culture medium was replenished with fresh medium containing RANKL and various treatments (SEC2 2M‐118 or conditioned medium) every 2 days. Trap staining was performed on day 5 to identify the effect of various treatments on osteoclast differentiation. Total RNAs were harvested from the cells to determine the osteoclastic gene expression profiles.

### Statistical Analysis

All the experiments were repeated at least 3 times. All results were shown as mean ± standard error. Statistical analysis was performed with GraphPad Prism 6.0 software (GraphPad Software, San Diego, CA, USA). Comparisons between two groups were performed using an unpaired two‐tailed Student's *t*‐test. Multiple group comparisons were analyzed using one‐way analysis of variance (ANOVA) with Bonferroni's post hoc. For comparisons of different treatments across multiple time points, two‐way ANOVA with Bonferroni's post hoc was used. *p* value < 0.05 was considered statistically significant.

## Conflict of Interest

Authors C.L., Y.J., J.C., and H.L. are employed by the company Shenyang Xiehe Biopharmaceutical Co. Ltd. The other authors declare that the research was conducted in the absence of any commercial or financial relationships that could be construed as a potential conflict of interest.

## Author Contributions

H.W. and S.L. contributed equally to this work. H.W. performed experiment and wrote the paper; S.L. refined the idea, performed experiment, and analyzed data. J.C., H.L. designed the study; L.F., X.L., Z.Y., Z.J., Y.‐C.L. X.Z., M.W., B.W., L.K., Q.P., S.B., Y.L., and Y.Y. provided technical assistance or performed part of the experiment; H.W., B.H. performed bioinformatics analysis; C.L. and Y.J. provided reagents and paper revising; W.Y.W.L., P.D.C., and M.D.T. contributed to paper revising. G.L. designed and supervised the project, obtained funding, and revised the paper.

## Supporting information

Supporting Information

Supplemental Table 1

## Data Availability

The data that support the findings of this study are available from the corresponding author upon reasonable request.

## References

[1] D. Ragipoglu , A. Dudeck , M. Haffner‐Luntzer , M. Voss , J. Kroner , A. Ignatius , V. Fischer , Front. Immunol. 2020, 11, 163. 32117297 10.3389/fimmu.2020.00163 PMC7025484

[2] L. Arboleya , S. Castaneda , Reumatol. Clin. 2013, 9, 303. 23727459 10.1016/j.reuma.2013.02.008

[3] M. Tsukasaki , H. Takayanagi , Nat. Rev. Immunol. 2019, 19, 626. 31186549 10.1038/s41577-019-0178-8

[4] M. F. Faienza , A. Ventura , F. Marzano , L. Cavallo , Clin. Dev. Immunol. 2013, 2013, 575936. 23762093 10.1155/2013/575936 PMC3677008

[5] Y. Li , G. Toraldo , A. Li , X. Yang , H. Zhang , W. P. Qian , M. N. Weitzmann , Blood 2007, 109, 3839. 17202317 10.1182/blood-2006-07-037994 PMC1874582

[6] S.‐K. Lee , Y. Kadono , F. Okada , C. Jacquin , B. Koczon‐Jaremko , G. Gronowicz , D. J. Adams , H. L. Aguila , Y. Choi , J. A. Lorenzo , J. Bone Miner. Res. 2006, 21, 1704. 17002560 10.1359/jbmr.060726

[7] M. Khass , H. Rashid , P. D. Burrows , A. Javed , H. W. Schroeder , Front. Immunol. 2022, 13, 906649. 36189270 10.3389/fimmu.2022.906649 PMC9516392

[8] R. K. Srivastava , H. Y. Dar , P. K. Mishra , Front. Immunol. 2018, 9, 657. 29675022 10.3389/fimmu.2018.00657 PMC5895643

[9] N. Wyzga , S. Varghese , S. Wikel , E. Canalis , F. A. Sylvester , Bone 2004, 35, 614. 15336596 10.1016/j.bone.2004.04.022

[10] M. Croes , F. C. Oner , D. van Neerven , E. Sabir , M. C. Kruyt , T. J. Blokhuis , W. J. A. Dhert , J. Alblas , Bone 2016, 84, 262. 26780388 10.1016/j.bone.2016.01.010

[11] L. Rifas , S. Arackal , M. N. Weitzmann , J. Cell. Biochem. 2003, 88, 650. 12577299 10.1002/jcb.10436

[12] S. Cenci , G. Toraldo , M. N. Weitzmann , C. Roggia , Y. Gao , W. P. Qian , O. Sierra , R. Pacifici , Proc. Natl. Acad. Sci. USA 2003, 100, 10405. 12923292 10.1073/pnas.1533207100 PMC193574

[13] S. Cenci , M. N. Weitzmann , C. Roggia , N. Namba , D. Novack , J. Woodring , R. Pacifici , J. Clin. Invest. 2000, 106, 1229. 11086024 10.1172/JCI11066 PMC381439

[14] Y. Gao , F. Grassi , M. R. Ryan , M. Terauchi , K. Page , X. Yang , M. N. Weitzmann , R. Pacifici , J. Clin. Invest. 2007, 117, 122. 17173138 10.1172/JCI30074 PMC1697800

[15] Y. Gao , W. P. Qian , K. Dark , G. Toraldo , A. S. Lin , R. E. Guldberg , R. A. Flavell , M. N. Weitzmann , R. Pacifici , Proc. Natl. Acad. Sci. USA 2004, 101, 16618. 15531637 10.1073/pnas.0404888101 PMC534514

[16] C. Roggia , Y. Gao , S. Cenci , M. N. Weitzmann , G. Toraldo , G. Isaia , R. Pacifici , Proc. Natl. Acad. Sci. USA 2001, 98, 13960. 11717453 10.1073/pnas.251534698 PMC61149

[17] J. H. An , H. Park , J. A. Song , K. H. Ki , J. Y. Yang , H. J. Choi , S. W. Cho , S. W. Kim , S. Y. Kim , J. J. Yoo , W. Y. Baek , J. E. Kim , S. J. Choi , W. Oh , C. S. Shin , Tissue Eng., Part A 2013, 19, 685. 23215868 10.1089/ten.tea.2012.0047 PMC3568969

[18] I. V. Pinchuk , E. J. Beswick , V. E. Reyes , Toxins 2010, 2, 2177. 22069679 10.3390/toxins2082177 PMC3153290

[19] W. Zhao , Y. Li , W. Liu , D. Ding , Y. Xu , L. Pan , S. Chen , Toxins 2016, 8, 185. 27322320 10.3390/toxins8060185 PMC4926151

[20] D. Etter , J. Schelin , M. Schuppler , S. Johler , Toxins 2020, 12, 584. 32927913 10.3390/toxins12090584 PMC7551944

[21] N. Labrecque , J. Thibodeau , R. P. Sekaly , Semin. Immunol. 1993, 5, 23. 8467091 10.1006/smim.1993.1004

[22] R. R. Rich , J. A. Mollick , R. G. Cook , Trans. Am. Clin. Climatol. Assoc. 1990, 101, 195. 2577245 PMC2376496

[23] J. Xu , T. Wu , Y. Sun , B. Wang , J. Zhang , W. Y. Lee , Y. Chai , G. Li , J. Orthop. Res. 2017, 35, 1215. 27431811 10.1002/jor.23372

[24] T. Wu , J. Zhang , B. Wang , Y. Sun , Y. Liu , G. Li , Bone Jt. Res. 2018, 7, 179. 10.1302/2046-3758.72.BJR-2017-0229.R1 PMC5895947 29682284

[25] J. Zhang , Y. M. Cai , M. K. Xu , Z. H. Song , C. Y. Li , H. R. Wang , H. H. Dai , Z. P. Zhang , C. X. Liu , Pharmazie 2013, 68, 359. 23802434

[26] M. Xu , X. Wang , Y. Cai , H. Zhang , H. Yang , C. Liu , C. Zhang , Cancer Immunol., Immunother. 2011, 60, 705. 21331815 10.1007/s00262-011-0986-6 PMC11028788

[27] J. E. Darnell Jr. , I. M. Kerr , G. R. Stark , Science 1994, 264, 1415. 8197455 10.1126/science.8197455

[28] X. Liu , L. Ye , Y. Bai , H. Mojidi , N. E. Simister , X. Zhu , J. Immunol. 2008, 181, 449. 18566411 10.4049/jimmunol.181.1.449 PMC2667120

[29] Z. Jin , J. Kho , B. Dawson , M. M. Jiang , Y. Chen , S. Ali , L. C. Burrage , M. Grover , D. J. Palmer , D. L. Turner , P. Ng , S. C. Nagamani , B. Lee , J. Clin. Invest. 2021, 131, e138935. 33373331 10.1172/JCI138935 PMC7919726

[30] M. Chen , H. Y. Sun , S. J. Li , M. Das , J. M. Kong , T. M. Gao , Neurosignals 2009, 17, 162. 19258725 10.1159/000205525

[31] D. D. Browning , M. P. McShane , C. Marty , R. D. Ye , J. Biol. Chem. 2000, 275, 2811. 10644746 10.1074/jbc.275.4.2811

[32] G. Duque , D. C. Huang , N. Dion , M. Macoritto , D. Rivas , W. Li , X. F. Yang , J. Li , J. Lian , F. T. Marino , J. Barralet , V. Lascau , C. Deschenes , L.‐G. Ste‐Marie , R. Kremer , J. Bone Miner. Res. 2011, 26, 1472. 21308779 10.1002/jbmr.350

[33] S. Khosla , L. C. Hofbauer , Lancet Diabetes Endocrinol. 2017, 5, 898. 28689769 10.1016/S2213-8587(17)30188-2 PMC5798872

[34] J. Zhang , K. G. Saag , J. R. Curtis , Clin. Rheum. Dis. 2011, 37, 387. 10.1016/j.rdc.2011.08.001 PMC4420195 22023898

[35] O. Lamy , D. Stoll , B. Aubry‐Rozier , E. G. Rodriguez , Curr. Osteoporosis Rep. 2019, 17, 8. 10.1007/s11914-019-00502-4 30659428

[36] N. R. Jorgensen , P. Schwarz , Curr. Osteoporosis Rep. 2011, 9, 149. 10.1007/s11914-011-0065-0 21698357

[37] W. M. Fu , X. Zhu , H. Wang , W. Wei‐Mao , J. Y. Chen , Y. Liang , J. F. Zhang , H. F. Kung , Exp. Cell Res. 2014, 322, 202. 24355808 10.1016/j.yexcr.2013.12.008

[38] S. Zhou , G. Wang , L. Qiao , Q. Ge , D. Chen , Z. Xu , D. Shi , J. Dai , J. Qin , H. Teng , Q. Jiang , Exp. Ther. Med. 2018, 15, 3623. 29545892 10.3892/etm.2018.5839 PMC5841068

[39] J. Chen , H. Zhang , X. Wu , F. Wang , Y. Wang , Q. Gao , H. Liu , Y. Hu , J. Su , Y. Jing , Stem Cells Int. 2021, 2021, 8546739. 34976071 10.1155/2021/8546739 PMC8720025

[40] M. N. Weitzmann , R. Pacifici , Ann. N. Y. Acad. Sci. 2007, 1116, 360. 18083938 10.1196/annals.1402.068

[41] L. Zhu , F. Hua , W. Ding , K. Ding , Y. Zhang , C. Xu , Immun. Ageing 2020, 17, 30. 33072163 10.1186/s12979-020-00202-z PMC7557094

[42] Z. S. Buchwald , J. R. Kiesel , R. DiPaolo , M. S. Pagadala , R. Aurora , PLoS One 2012, 7, e38199. 22701612 10.1371/journal.pone.0038199 PMC3368916

[43] X. Qiu , Y. Gui , N. Zhang , Y. Xu , D. Li , L. Wang , BioSci. Trends 2016, 10, 400. 27476527 10.5582/bst.2016.01012

[44] M. A. Garcia‐Perez , I. Noguera , C. Hermenegildo , A. Martinez‐Romero , J. J. Tarin , A. Cano , Hum. Reprod. 2006, 21, 880. 16459351 10.1093/humrep/dei413

[45] L. Song , Y. N. Bi , P. Y. Zhang , X. M. Yuan , Y. Liu , Y. Zhang , J. Y. Huang , K. Zhou , BioMed Res. Int. 2017, 2017, 8417814. 29119115 10.1155/2017/8417814 PMC5651096

[46] D. Cakouros , S. Hemming , K. Gronthos , R. Liu , A. Zannettino , S. Shi , S. Gronthos , Epigenet. Chromatin 2019, 12, 3. 10.1186/s13072-018-0247-4 PMC6317244 30606231

[47] S. J. Jackson , N. Andrews , D. Ball , I. Bellantuono , J. Gray , L. Hachoumi , A. Holmes , J. Latcham , A. Petrie , P. Potter , A. Rice , A. Ritchie , M. Stewart , C. Strepka , M. Yeoman , K. Chapman , Lab. Anim. 2017, 51, 160. 27307423 10.1177/0023677216653984 PMC5367550

[48] V. Glatt , E. Canalis , L. Stadmeyer , M. L. Bouxsein , J. Bone Miner. Res. 2007, 22, 1197. 17488199 10.1359/jbmr.070507

[49] J. Zhang , Q. Fu , Z. Ren , Y. Wang , C. Wang , T. Shen , G. Wang , L. Wu , Gynecol. Endocrinol. 2015, 31, 183. 25384921 10.3109/09513590.2014.975683

[50] W. Zhang , W. Zhao , W. Li , Q. Geng , R. Zhao , Y. Yang , L. Lv , W. Chen , Front. Endocrinol. 2022, 13, 779264. 10.3389/fendo.2022.779264 PMC9205399 35721756

[51] V. Breuil , M. Ticchioni , J. Testa , C. H. Roux , P. Ferrari , J. P. Breittmayer , C. Albert‐Sabonnadiere , J. Durant , F. De Perreti , A. Bernard , L. Euller‐Ziegler , G. F. Carle , Osteoporosis Int. 2010, 21, 805. 10.1007/s00198-009-1018-7 19876583

[52] G. Duque , D. C. Huang , M. Macoritto , D. Rivas , X. F. Yang , L. G. Ste‐Marie , R. Kremer , Stem Cells 2009, 27, 550. 19096039 10.1634/stemcells.2008-0886

[53] T. Maruhashi , T. Kaifu , R. Yabe , A. Seno , S. H. Chung , N. Fujikado , Y. Iwakura , J. Immunol. 2015, 194, 5681. 25926676 10.4049/jimmunol.1500273

[54] R. J. van't Hof , S. H. Ralston , Immunology 2001, 103, 255. 11454054 10.1046/j.1365-2567.2001.01261.x PMC1783253

[55] J. Joshua , H. Kalyanaraman , N. Marathe , R. B. Pilz , Vitam. Horm. 2014, 96, 247. 25189390 10.1016/B978-0-12-800254-4.00010-6

[56] J. Klein‐Nulend , R. F. van Oers , A. D. Bakker , R. G. Bacabac , Osteoporosis Int. 2014, 25, 1427. 10.1007/s00198-013-2590-4 24322479

[57] S. J. Wimalawansa , G. De Marco , P. Gangula , C. Yallampalli , Bone 1996, 18, 301. 8726385 10.1016/8756-3282(96)00005-1

[58] Y. Liu , L. Wang , T. Kikuiri , K. Akiyama , C. Chen , X. Xu , R. Yang , W. Chen , S. Wang , S. Shi , Nat. Med. 2011, 17, 1594. 22101767 10.1038/nm.2542 PMC3233650

[59] A. Herman , G. Croteau , R. P. Sekaly , J. Kappler , P. Marrack , J. Exp. Med. 1990, 172, 709. 2117633 10.1084/jem.172.3.709 PMC2188560

[60] M. I. Love , W. Huber , S. Anders , Genome Biol. 2014, 15, 550. 25516281 10.1186/s13059-014-0550-8 PMC4302049

