# Supplementary material for: Low‐Dose Staphylococcal Enterotoxin C2 Mutant Maintains Bone Homeostasis via Regulating Crosstalk between Bone Formation and Host T‐Cell Effector Immunity
Source: Adv Sci (Weinh). 2023 Aug 8;10(28):2300989. doi: 10.1002/advs.202300989 (PMC10558680; doi:10.1002/advs.202300989)
Supplement: Supplementary file 1 — Supporting Information [file ADVS-10-2300989-s002.pdf]

ADVANCED SCIENCE
Open Access

Supporting Information

for *Adv. Sci.*, DOI 10.1002/advs.202300989

Low-Dose Staphylococcal Enterotoxin C2 Mutant Maintains Bone Homeostasis via Regulating Crosstalk between Bone Formation and Host T-Cell Effector Immunity

Haixing Wang, Sien Lin, Lu Feng, Baozhen Huang, Xuan Lu, Zhengmeng Yang, Zhaowei Jiang, Yu-Cong Li, Xiaoting Zhang, Ming Wang, Bin Wang, Lingchi Kong, Qi Pan, Shanshan Bai, Yuan Li, Yongkang Yang, Wayne Yuk Wai Lee, Peter D. Currie, Changshuang Lin, Yanfu Jiang, Juyu Chen, Micky D. Tortorella, Hongyi Li* and Gang Li**

Supporting Information

Low-dose Staphylococcal Enterotoxin C2 Mutant maintains Bone Homeostasis via regulating Crosstalk between Bone Formation and Host T cells Effector Immunity

Haixing Wang†, Sien Lin†, Lu Feng, Baozhen Huang, Zhengmeng Yang, Xiaoting Zhang, Ming Wang, Bin Wang, Lingchi Kong, Qi Pan, Yu-cong Li, Shanshan Bai, Xuan Lu, Yuan Li, Zhaowei Jiang, Yongkang Yang, Wayne Yuk Wai Lee, Peter D. Currie, Changshuang Lin, Yanfu Jiang, Juyu Chen, Micky D. Tortorella Hongyi Li*, Gang Li**

Supplementary Figures

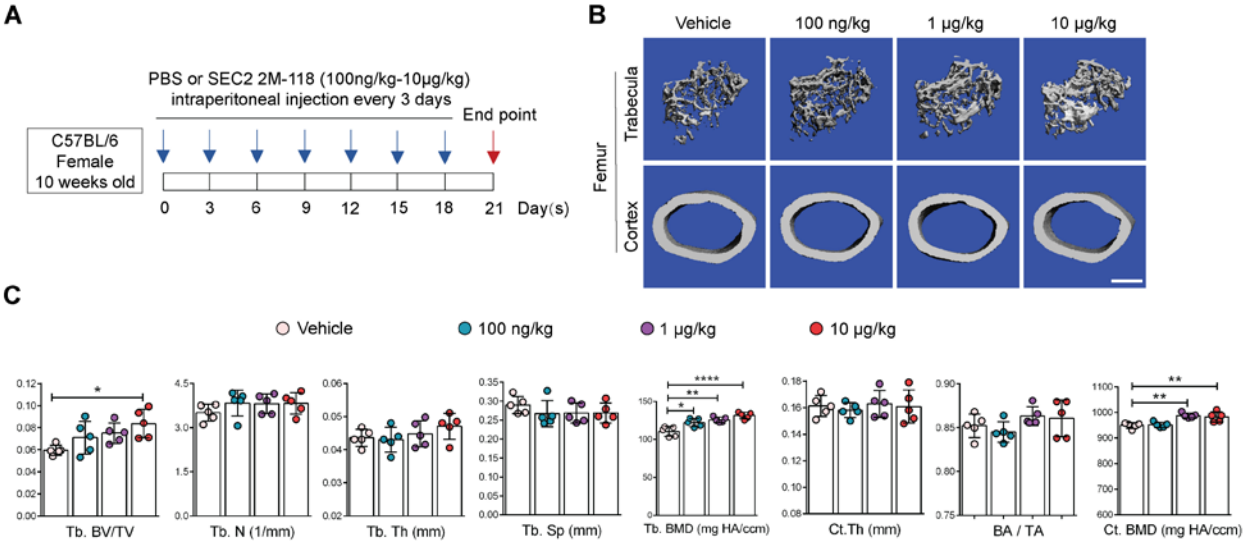

Figure S1. Systemic administration of SEC2 2M-118 increases bone mass in normal mice. A) Different doses of SEC2 2M-118 were given by intraperitoneal injection in normal C57BL/6 mice for 3 weeks. B) 3-D reconstruction of trabecular bone in distal femur and cortical bone in midshaft of femur from microcomputed tomography (μCT) scans. Scale bar, 500 μm. C) Quantitative analyses of microstructural parameters of bone including bone volume/tissue volume (BV/TV), trabecular number (Tb. N), trabecular thickness (Tb. Th), trabecular separation (Tb. sp), trabecular bone mineral density (Tb. BMD), cortical bone thickness (Ct. Th), bone area/tissue area (BA/TA) and cortical bone mineral density (Ct. BMD). n=5. Data were shown as Mean ± SD. One-way ANOVA was used with Bonferroni multiple comparisons test. *$p<0.05$, ** $p<0.01$, **** $p<0.0001$.

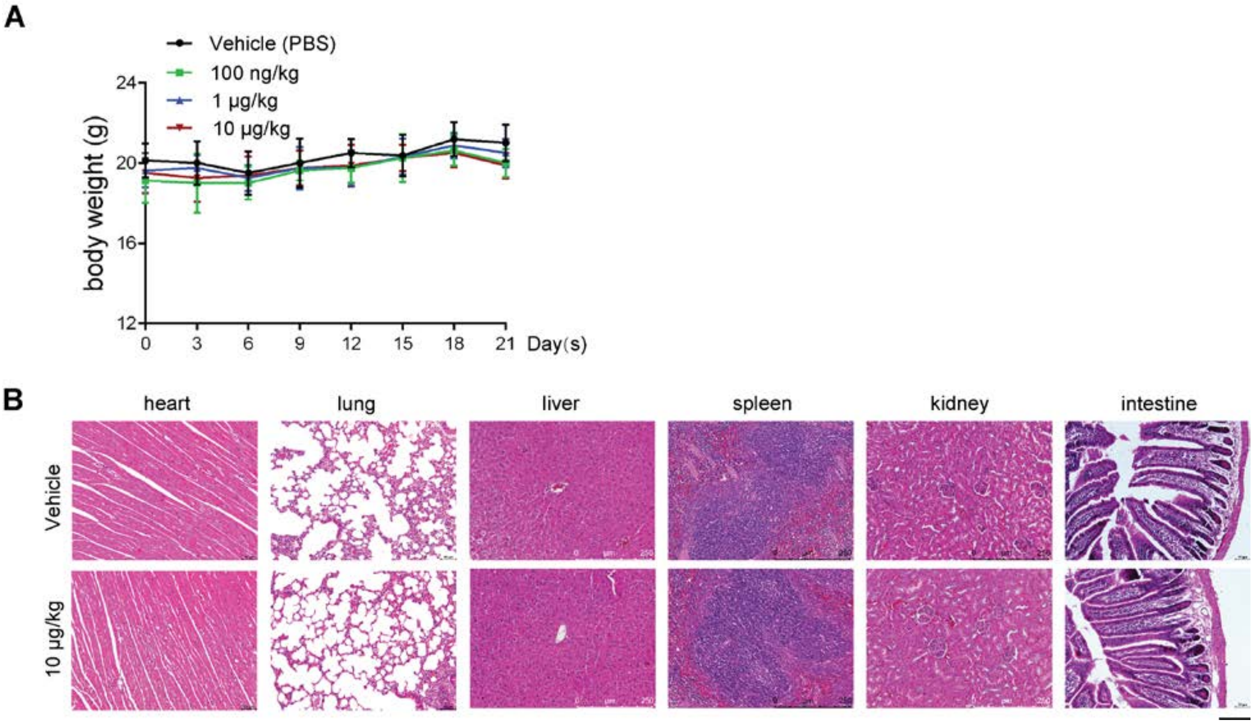

Figure S2. Administration of SEC2 2M-118 *in vivo* shows no notable toxicity. A) Mice were given various dosages (100 ng/kg, 1 µg/kg, and 10 µg/kg) of SEC2 2M-118 by intraperitoneal injections every 3 days, and body weight was determined before each injection. *n*=4 for each group. Data was shown as Mean ± SD. B) After systemic administration of SEC2 2M-118 (10 µg/kg) for 3 weeks, major organs including heart, lung, liver, kidney, spleen, and intestine were collected for histologic investigations. *n*=3 for each group. Scale bar, 100 µm.

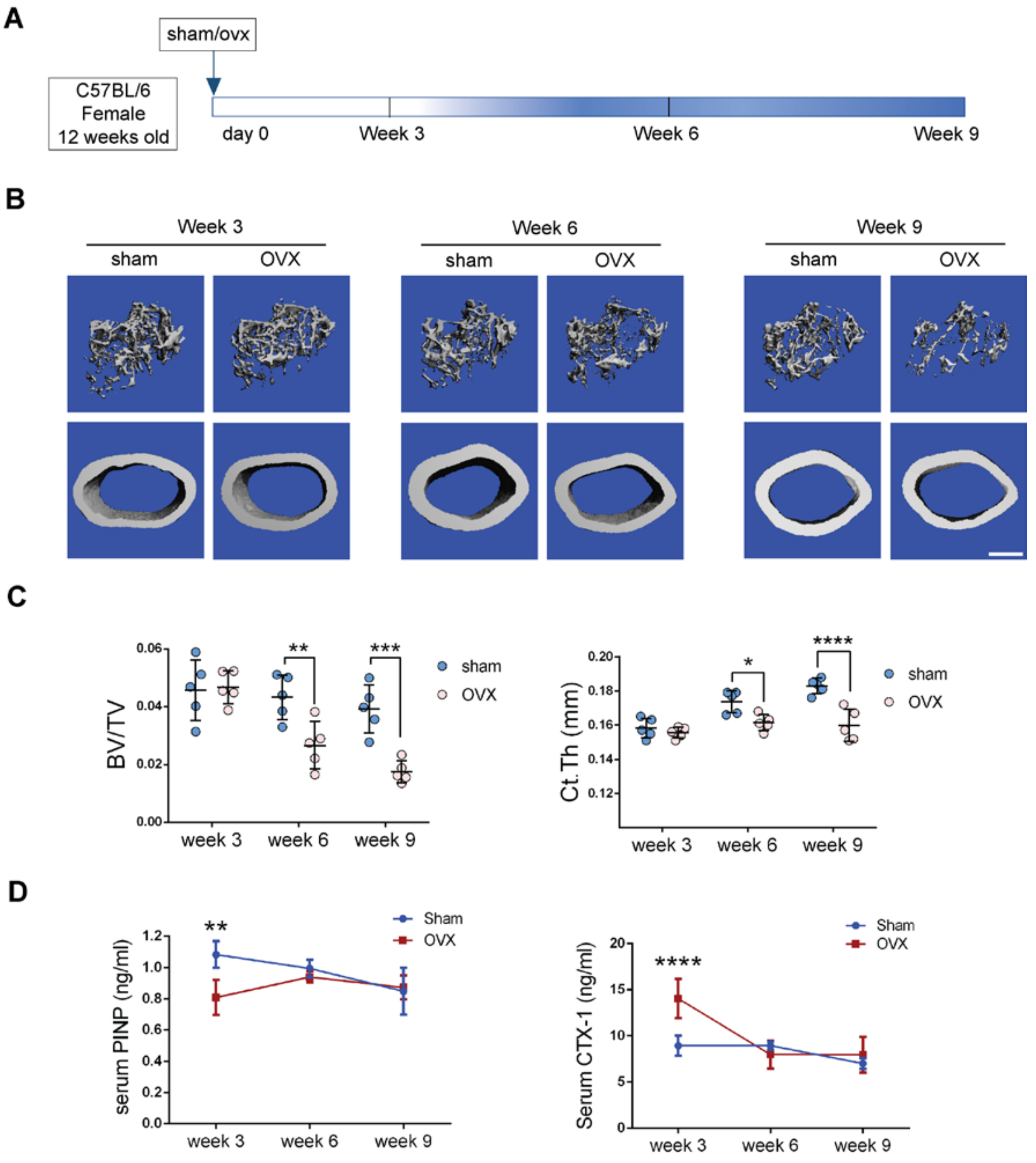

Figure S3. Dynamic change of bone quality and bone turnover markers post OVX. A) 12-week-old C57BL/6 mice received OVX surgery. Dynamic change of bone structure was evaluated at week 3, week 6 and week 9 after OVX surgery. B, C**)** Micro-CT analyses of trabecular and cortical bone of femur. The bone volume/tissue volume of trabecular bone and thickness of femur midshaft were present. *n*=5. Scale bar in (B), 500 µm. D) Serum levels of PINP and CTX-1 at different time points post OVX. *n*=4-5. Data was shown as Mean ± SD. Two-way ANOVA was used with Bonferroni multiple comparisons test. **p*<0.05, ** *p*<0.01, *** *p*<001, **** *p*<0.0001.

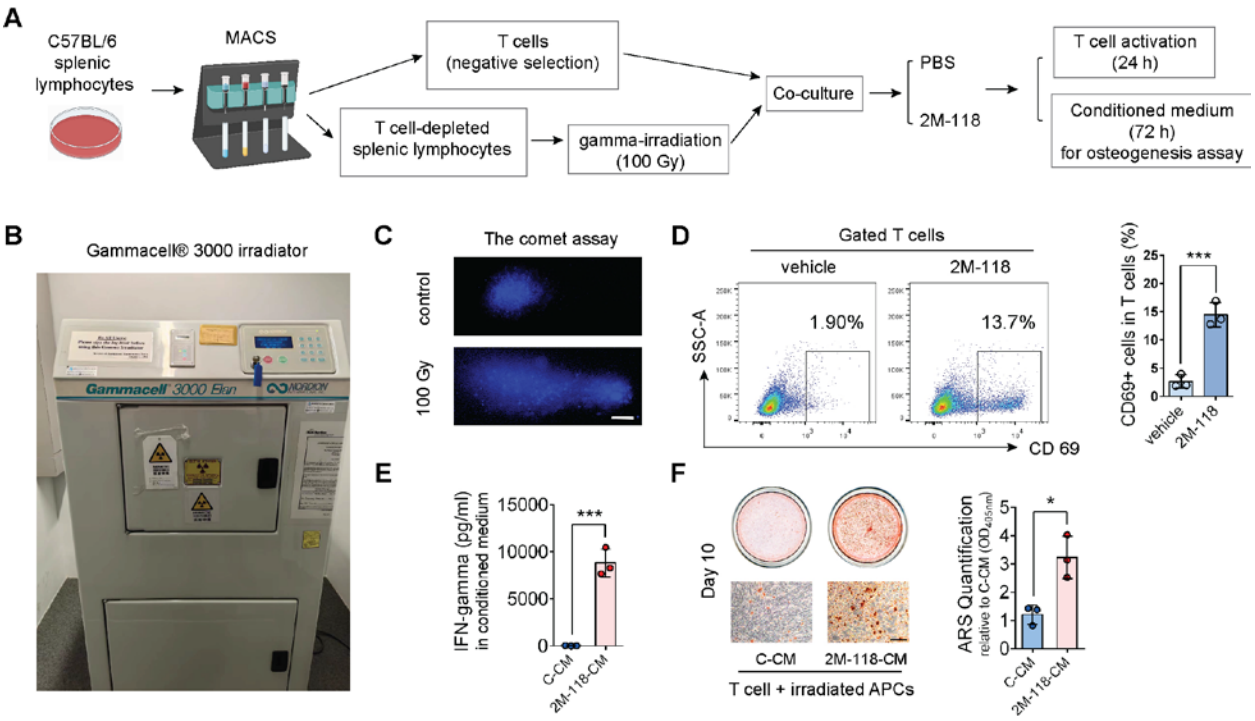

Figure S4. Purified T cells cocultured with autologous irradiated APCs show similar responses to 2M-118 stimulation compared to splenic lymphocytes. A) T cell-depleted splenic cells (Antigen presenting cells, APCs) were isolated by MACS and then received gamma irradiation (100 Gy) in GC 3000 irradiator. Purified T cells were cocultured with the autologous irradiated splenic cells and stimulated with or without 2M-118 for 24 or 72 hours. T cell activation marker CD 69 was analyzed and conditioned medium was collected for following osteogenesis assays. B) Photo showing the radiation apparatus GC 3000 for cell irradiation. C) Verification of DNA damage after gamma irradiation using comet assay. Alkaline lysis and electrophoresis were performed, and DNA was stained with DAPI for imaging. Scale bar in (C), 5 µm. D) T cell activation was analyzed after 2M-118 stimulation for 24 hours with the presence of irradiated APCs. *n*=3. E) Concentrations of IFN-γ in conditioned medium prepared from enriched T cell with irradiated APCs after 2M-118 stimulation were tested by ELISA. *n*=3. F) MC-3T3-E1 cells were induced to differentiate into osteoblasts for 10 days. C-CM (10%) or 2M-118-CM (10%) prepared from enriched T cells with irradiated APCs was added into the differentiation medium. Quantification of Alizarin red staining showing the effect of the conditioned medium on osteogenesis. *n*=3. Scale bar, 100 µm. Data were shown as Mean ± SD. Student's t-test was used to compare parameters between two groups. *p<0.05. *** p<001.

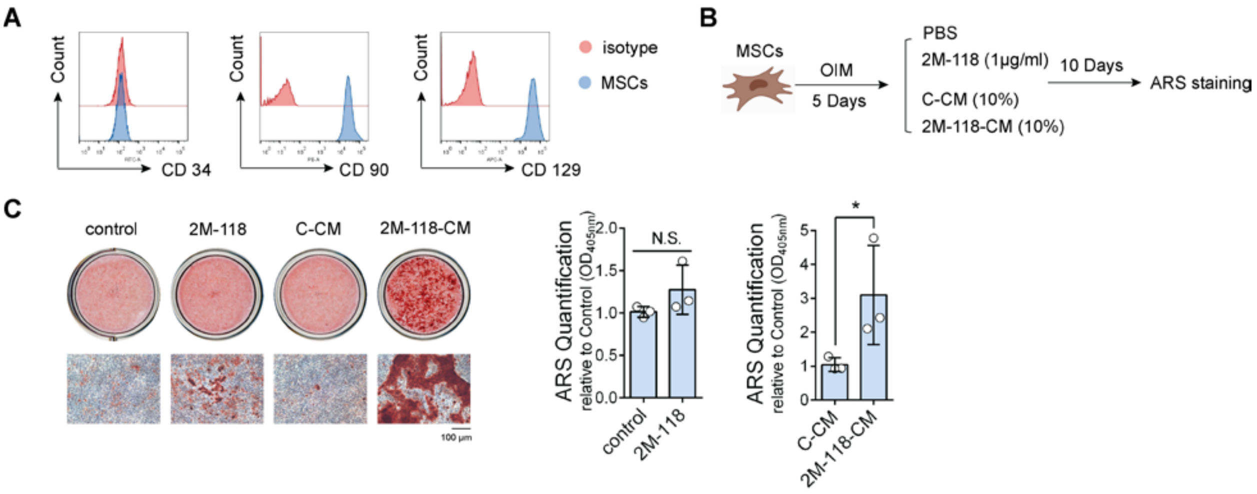

Figure S5. Conditioned medium from SEC2 2M-118 stimulated splenic lymphocytes promotes osteogenesis from rat bone marrow MSCs. A) Identification of MSC markers (negative marker CD34, positive marker CD90 and CD129). B) MSCs were treated with osteogenic induction medium (OIM) for 5 days, then PBS/2M-118 or conditioned medium (C-CM/2M-118-CM) was added into the culture medium for another 10 days' stimulation. C) Quantification of Alizarin red staining showed conditioned medium from SEC2 2M-118 stimulated mixed lymphocytes promoted osteogenesis from rat bone marrow MSCs. *n*=3. Scale bar, 100 μm. Data were shown as Mean ± SD. Student's t-test was used to compare parameters between two groups. *p<0.05. N.S.: not significant.

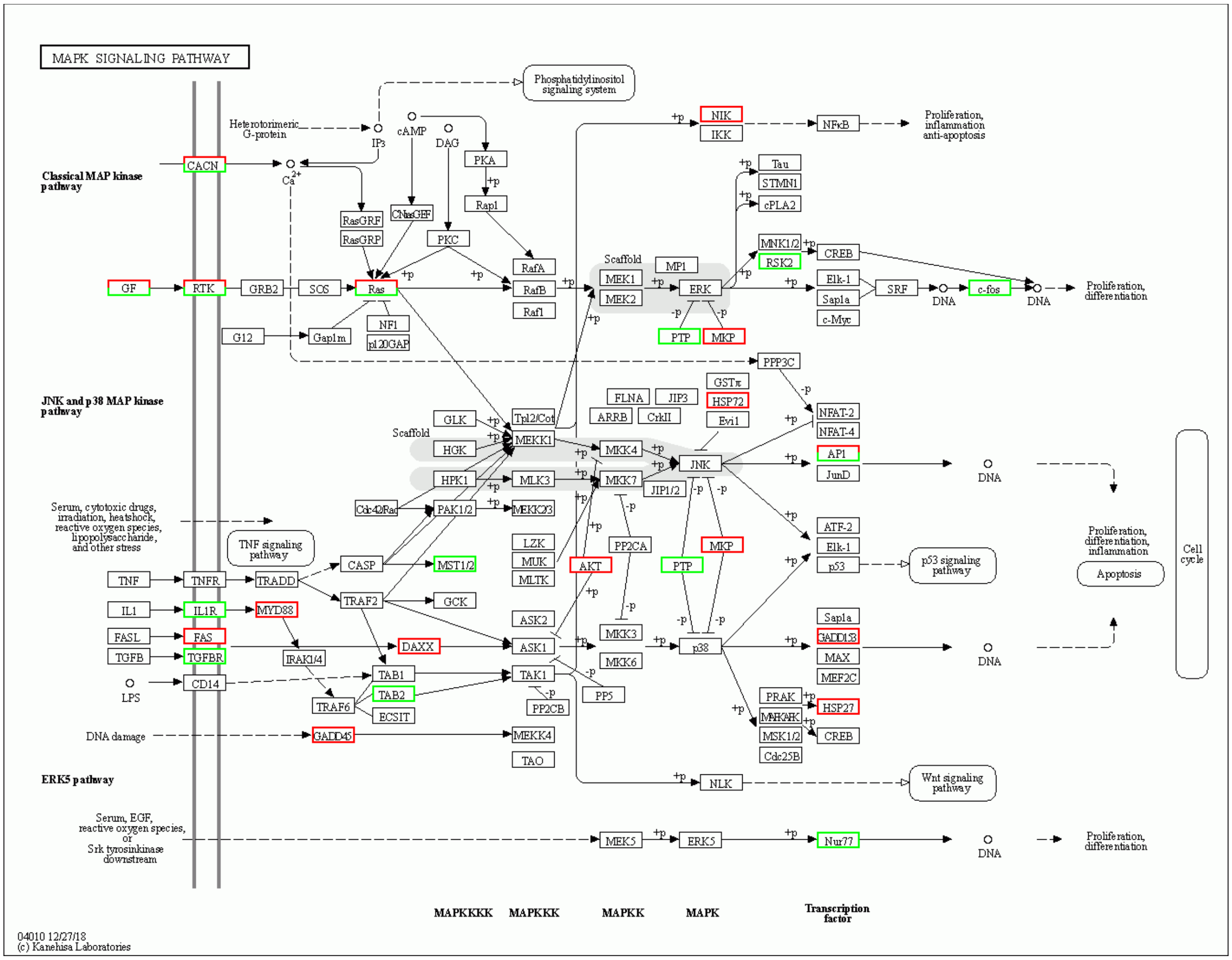

Figure S6. RNA-sequencing analysis showing the effects of IFN-γ on MAPK signaling pathways. Red boxes highlight upregulated genes, while green boxes indicate down-regulated genes. (DEGs were defined as Q value⩽0.05. No limitation of fold change)

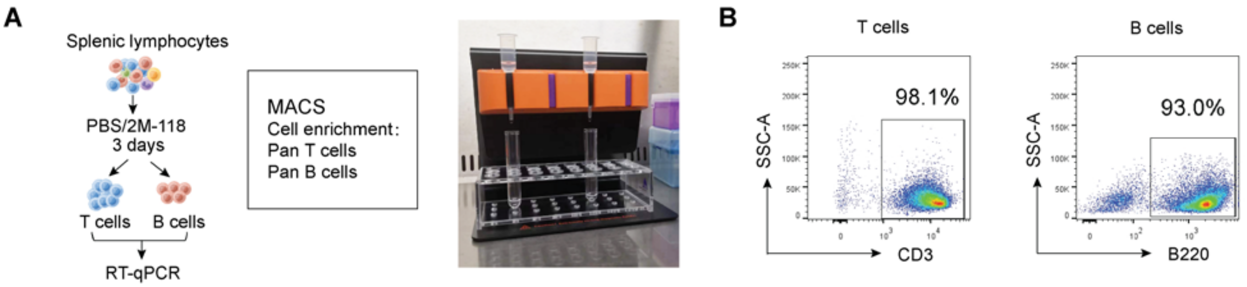

Figure S7. Isolation of T cells and B cells with MACS negative selection. A) Photo showing the MACS cell separator used for T cells and B cells enrichment from 2M-118 stimulated splenic lymphocytes. B) Flow cytometry analysis showing the purity of T cells and B cells after MACS negative selection.

Tables S1

Table S1 Primer sequences for quantitative real-time PCR

Gene	NCBI Gene ID	Primer sequences
Bglap (Osteocalcin, Ocn)	12096	Forward 5'-CTGACCTCACAGATCCCAAGC-3'
		Reverse 5'- TGGTCTGATAGCTCGTCACAAG-3'
Ctsk (Cathepsin K)	13038	Forward 5'-CTTCCAATACGTGCAGCAGA-3'
		Reverse 5'-TCTTCAGGGCTTTCTCGTTC-3'
Il1b (IL-1β)	16176	Forward 5'- TTCAGGCAGGCAGTATCACTC-3'
		Reverse 5'- GAAGGTCCACGGGAAAGACAC-3'
Il2	16183	Forward 5'- GTGCTCCTTGTCAACAGCG-3'
		Reverse 5'- GGGGAGTTTCAGGTTCCTGTA -3'
Il4	16189	Forward 5'- GGTCTCAACCCCCAGCTAGT-3'
		Reverse 5'- GCCGATGATCTCTCTCAAGTGAT-3'
Il6	16193	Forward 5'-TAGTCCTTCCTACCCCAATTTCC-3'
		Reverse 5'-TTGGTCCTTAGCCACTCCTTC-3'
Il10	16153	Forward 5'- GCTCTTACTGACTGGCATGAG-3'
		Reverse 5'- CGCAGCTCTAGGAGCATGTG-3'
Il17a	16171	Forward 5'-TCAGCGTGTCCAAACACTGAG-3'
		Reverse 5'-CGCCAAGGGAGTTAAAGACTT-3'
Tnf (TNF-a)	21926	Forward 5'-CCCTCACACTCAGATCATCTTCT-3'
		Reverse 5'-GCTACGACGTGGGCTACAG-3'
Ifng	15978	Forward 5'-ACAGCAAGGCGAAAAAGGATG-3'
		Reverse 5'-TGGTGGACCACTCGGATGA-3'
Ccl2	20296	Forward 5'-TTAAAAACCTGGATCGGAACCAA-3'
		Reverse 5'-GCATTAGCTTCAGATTTACGGGT-3'
Ccl3	20302	Forward 5'-TTCTCTGTACCATGACACTCTGC-3'
		Reverse 5'-CGTGGAATCTTCCGGCTGTAG-3'
Csf3 (G-CSF)	12985	Forward 5'-GCACTATGGTCAGGACGAGAG-3'
		Reverse 5'-GGGGAAATACCCGATAGAGCC-3'
Tgfb1	21803	Forward 5'-CTCCCGTGGCTTCTAGTGC-3'
		Reverse 5'-GCCTTAGTTTGGACAGGATCTG-3'
Bmp2	12156	Forward 5'-TCTTCCGGGAACAGATACAGG-3'
		Reverse 5'-TGGTGTCCAATAGTCTGGTCA-3'
Wnt10b	22410	Forward 5'-CGGACTGAGTAAGCGACAGC-3'
		Reverse 5'-ACTCGTGAACGGCGATGTG-3'
Tnfsf11 (RANKL)	21943	Forward 5'- AGCCGAGACTACGGCAAGTA-3'
		Reverse 5'- AAAGTACAGGAACAGAGCGATG-3'
Tnfrsf11b (osteoprotegerin, Opg)	18383	Forward 5'- GGGCGTTACCTGGAGATCG-3'
		Reverse 5'- CGTTGTCATGTGTTGCATTTCC-3'
Nos2 (iNOS)	18126	Forward 5'- GTTCTCAGCCCAACAATACAAGA-3'
		Reverse 5'-GTGGACGGGTCGATGTCAC-3'

Gapdh	14433	Forward 5’- ACTTTGTCAAGCTCATTTCC -3’
		Reverse 5’- TGCAGCGAACTTTATTGATG -3’
